# Known structure, unknown function: An inquiry‐based undergraduate biochemistry laboratory course

**DOI:** 10.1002/bmb.20873

**Published:** 2015-07-06

**Authors:** Cynthia Gray, Carol W. Price, Christopher T. Lee, Alison H. Dewald, Matthew A. Cline, Charles E. McAnany, Linda Columbus, Cameron Mura

**Affiliations:** ^1^ Department of ChemistryUniversity of VirginiaCharlottesvilleVirginia22904; ^2^ Christopher T. Lee is currently at: Department of Chemistry & BiochemistryUniversity of California San DiegoLa JollaCalifornia92093; ^3^ Alison H. Dewald is currently at: Department of ChemistrySalisbury UniversitySalisburyMaryland21801

**Keywords:** biochemistry laboratory, protein function, functional genomics, inquiry‐based learning, active learning, curriculum, undergraduate research

## Abstract

Undergraduate biochemistry laboratory courses often do not provide students with an authentic research experience, particularly when the express purpose of the laboratory is purely instructional. However, an instructional laboratory course that is inquiry‐ and research‐based could simultaneously impart scientific knowledge and foster a student's research expertise and confidence. We have developed a year‐long undergraduate biochemistry laboratory curriculum wherein students determine, via experiment and computation, the function of a protein of known three‐dimensional structure. The first half of the course is inquiry‐based and modular in design; students learn general biochemical techniques while gaining preparation for research experiments in the second semester. Having learned standard biochemical methods in the first semester, students independently pursue their own (original) research projects in the second semester. This new curriculum has yielded an improvement in student performance and confidence as assessed by various metrics. To disseminate teaching resources to students and instructors alike, a freely accessible *Biochemistry Laboratory Education* resource is available at http://biochemlab.org. © 2015 The Authors Biochemistry and Molecular Biology Education published by Wiley Periodicals, Inc. on behalf of International Union of Biochemistry and Molecular Biology, 43(4):245–262, 2015.

**Abbreviations**
3D: three‐dimensional
GM: group meeting
IMAC: immobilized metal affinity chromatography
JCSG: Joint Center for Structural Genomics
LDH: lactate dehydrogenase
MSA: multiple sequence alignment
MW: molecular weight
PDB: Protein Data Bank
POI: protein of interest
SALG: student assessment of their learning gains
TOPSAN: The Open Protein Structure Annotation Network
UVa: University of Virginia

## Introductory Overview

The undergraduate biochemistry laboratories at the University of Virginia (UVa) have been redesigned as inquiry‐/research‐based laboratory courses taught across two semesters (Chem4411/4421, Biological Chemistry Labs I/II). This redesign was spurred by the need to have students engage in novel research in the context of an otherwise typical undergraduate laboratory course. The first semester of the new curriculum is dedicated to instruction in modern biochemical concepts and methods, including computational biology, while the second semester focuses on an authentic (publication‐grade) research question. Students apply the methods and concepts from the first semester to design and execute a functional assay of their protein of interest (POI) in the second semester. Each student's ultimate goal is to biochemically determine the function of their POI, for which the three‐dimensional (3D) structure has been determined and a putative function bioinformatically annotated (based on structure), but for which no experimental functional data exist. The year‐long course concludes with groups of students preparing a manuscript akin to a scientific paper and orally presenting a scientific poster that details their findings. If appropriate, the students' protein characterization results are disseminated as annotated entries in *The Open Protein Structure Annotation Network* (TOPSAN; http://www.topsan.org 1); thus far, nearly 10 *Biochemistry Laboratory Education* (BioLEd) POIs have been developed into new TOPSAN entries and, in ideal cases, student work has culminated in publications in the primary literature (e.g. 2).

**Figure 1:**
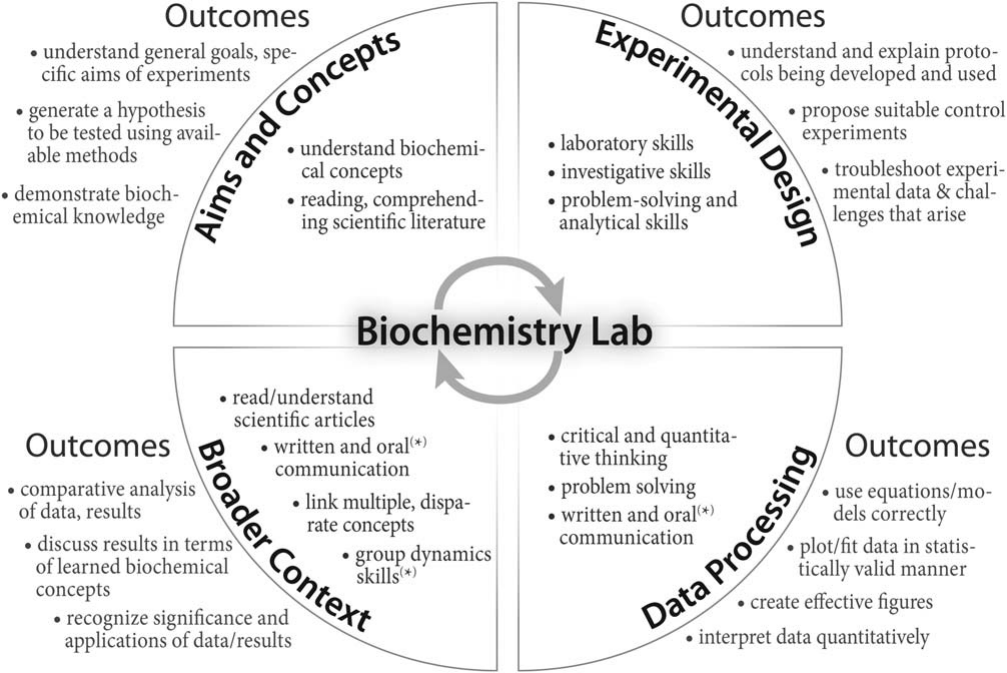
The four learning gains assessed as part of the BioLEd curriculum (bold font in each quadrant) encompass eight focal areas, with some level of redundancy. Asterisks denote those learning foci that are addressed most intensely in the second half of the full year‐long course.

**Figure 2:**
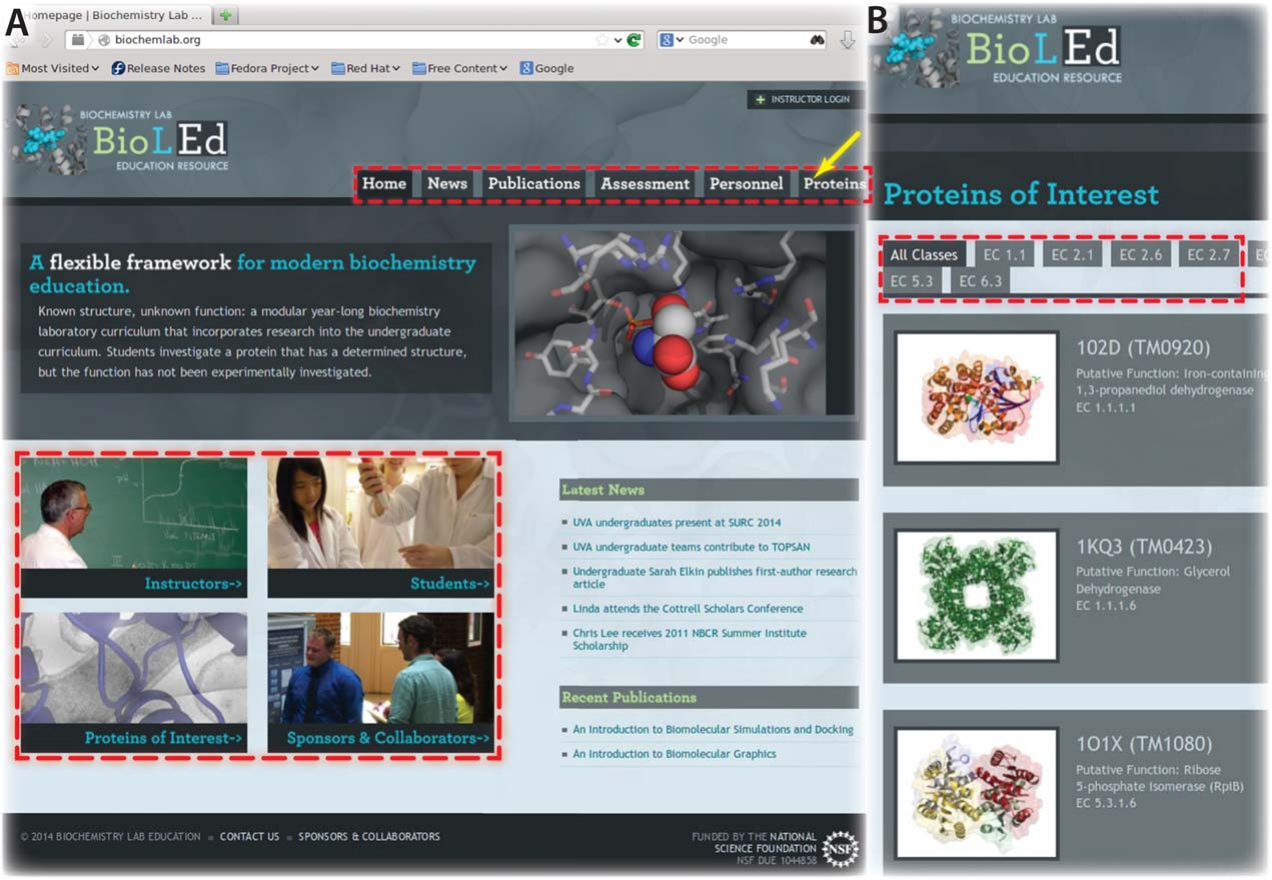
The BioLEd website, shown in this screenshot (*A*), features distinct portals (red boxes) for Students, Instructors, Collaborators, and Proteins of Interest. The Proteins tab (*B*; yellow arrow) opens a list of POIs, arranged by enzyme class, that have been investigated by students in current or past BioLEd labs. This resource has been built and maintained using a standards‐compliant content management system (WordPress), providing a modern and easily navigable framework for the BioLEd resource.

While the students focus on a well‐defined research goal, centered on functional characterization of their protein, our goals as instructors include teaching students (i) how to design and execute their own experiments, (ii) how to analyze data critically, (iii) how to work in a group towards a common goal, and (iv) how to communicate their work both orally and in writing (Fig. 1 and below). A further aim has been to create a *modular* curriculum that can be adopted by instructors at any college or university; a modular design affords instructors the option to focus on discrete portions of the curriculum, versus wholly implementing all of it. In addition to assessment of the new curriculum, our final goal has been broad dissemination of the course materials. A freely accessible BioLEd resource has been developed for this purpose at http://biochemlab.org (Fig. 2, and below).

## Motivation

Published reports and peer‐reviewed studies indicate that undergraduate science education must change from traditional, memorization‐based instruction to a more experience‐based form of learning 3, 4, 5, 6, 7. These studies find that students who engage in inquiry‐based learning develop better reasoning skills and more deeply enjoy research and laboratory work, versus students taught using traditional methods 8. While traditional laboratory courses often utilize the same *conceptual learning* style 9 that is typically used in lecture courses, this instructional style rarely encourages students to be independent/critical thinkers. In short, there is a demand for robust and accessible undergraduate science education curricula that provide more *experience‐based* learning, in a more active environment. To stimulate student autonomy and independence, such as would be required in a “real” research environment, laboratory courses must focus on teaching more *procedural knowledge* 9—including laboratory skills, experimental design, and data analysis and interpretation. Our new BioLEd curriculum employs both conceptual and procedural learning via guided instruction in the first semester (in the form of basic concepts, tools and protocols), and self– and peer–driven learning in the second semester (in the form of open‐ended experimental investigations, using the concepts, tools and methods from the first semester).

Historically, UVa's Biological Chemistry Laboratory courses had been taught in a conventional format: structured, single‐session laboratory exercises focused on teaching one technique via a procedure that had been validated by countless generations of prior classes. In Spring 2009, the traditional laboratory format for the second semester (Biochemistry Laboratory II) was abandoned in favor of a research‐based curriculum, taking as a starting point the myriad proteins of unknown function that have been structurally characterized in the past decade via structural genomics initiatives 10, 11, 12. Successive course modifications and adjustments to the curriculum followed, leading to our current year‐long course design. As the course has evolved, BioLEd's curricular design and logistics have been refined in accord with national calls for changes in undergraduate science education 3, 5, 13, 14. Across all iterations and refinement cycles, the goals of the laboratory course continue to be the same: to develop students' critical thinking skills, via hands‐on research, and to train them in methods used in the biochemistry workforce (Table 1). Students in the BioLEd curriculum engage in research from the point of inception onward. In the opening weeks of the first term, they learn bioinformatic methods and literature tools to enable them to formulate questions and hypotheses about their assigned target POI and its potential function (including what the word “function” can mean in various contexts). Throughout the second semester, students design and execute experimental plans; they perform enzyme kinetics studies and other experiments; they collect, process and interpret data; and they communicate their findings in oral and written form (their end‐of‐term manuscript is in the style of a scientific research article). The role of the instructors in this course, particularly during the research‐intensive second term, is to provide guidance and serve as a resource, and not to dictate the research steps directly. Here, we describe the new BioLEd initiative, which is inquiry‐based (first term) and research‐based (second term). We have defined precise learning gains for our modules (Fig. 1 and Supporting Information 1) in order to guide curricular design and refinement, and to assess student performance.

**Table 1:** First‐semester course modules

Week	Module	Assignment type
1a	Literature searches; electronic resources and tools (e.g. Pymol)	Problem set
1b	Basics of pipetting with the micropipette	Practical (wet‐laboratory)
2	Critically reading the primary literature (in‐laboratory discussion)	Problem set
3	Making biochemical buffers and solutions	Calculations
4	Enzyme kinetics (using LDH as a test system)	Laboratory report
5	Analyzing enzyme kinetics data (computer laboratory)	
6	Computational biology, I: Bioinformatic tools, web/database resources	Problem set
7a	General molecular cloning and transformation	Laboratory report
7b	Recombinant protein expression and SDS‐PAGE	
8	Protein purification, I: Gel‐filtration and ion‐exchange chromatography	
9	Protein purification, II: Affinity chromatography	
10	Quantitative protein concentration determination; ligand‐binding	Laboratory report
11	Computational biology, II: Molecular visualization, modeling, docking	POI report

BioLEd builds upon educational principles and best practices gleaned from other efforts over the past decade. For example, the merits of a modular approach have been recognized 15, as have the benefits of group‐based learning 16 and the necessity of introducing computational approaches into undergraduate biochemistry and molecular biology curricula 17. Also, other laboratory curricula that utilize both the expository‐ and inquiry‐based approaches have been recently developed (e.g. 18), and we are not alone in suggesting protein functional annotation as a means by which to introduce undergraduates to research 19. Appealing features of the BioLEd curriculum include: (i) its *functional genomics* framework, which leverages established biochemical methods to pursue open research questions of each POI's function; (ii) its fundamentally *modular* and *transferable* curriculum design, enabling facile adoption by other institutions/instructors; (iii) its *active learning* approaches, which pervade every aspect of the curriculum; (iv) its inclusion of *computational biology*, both informatics‐based and molecular (e.g. docking).

## Description of the Course

**Figure 3:**
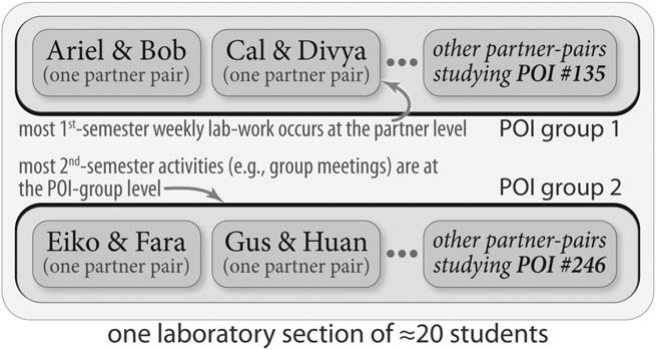
Group work is a core element of BioLEd's design and implementation. This schematic shows the relationship between an individual student and her laboratory partner (inner shell), other pairs (middle shell), and the higher‐order association of groups who work on the same POI in a laboratory section (outer shell); the two distinct POI groups in a typical ≈20‐student laboratory section are indicated. Within one full term, various assignments occur at either the individual level, partner level, or POI‐group level (work at the POI‐group level is chiefly in the second semester).

Throughout the year‐long course, students are charged with purifying and characterizing a protein for which the crystal structure was determined by the Joint Center for Structural Genomics (JCSG 20) and a putative function was annotated but never experimentally investigated. In order to optimize the chances of success and orchestrate course logistics, the experienced (PhD‐level) instructors select proteins of interest (POI) with presumed *enzymatic* functions and assign these to students (see the *Target Selection* section for sample criteria). Students learn a wide variety of techniques to study their assigned POI in the first semester, including bioinformatic and computational methods, extensive literature surveys, and laboratory experiments in which they over‐express, purify and quantify their recombinant POI. In addition, students learn how to determine enzyme kinetics via spectrophotometric assays, using the well‐characterized and commercially available enzyme lactate dehydrogenase (LDH). Apart from the LDH assay, each experiment represents truly unique and original research because each student POI group (Fig. 3) is working with a different, hitherto‐unexplored protein.

Students continuing in the second semester of the laboratory are already familiar with the techniques needed to study their POI. And, because of their literature mining and bioinformatic work, they possess much background knowledge about their unique protein. This preparation allows the second semester to be less rigidly structured than the first, which is also a necessity because each POI is unique; as is true of any scientific research, a “one size fits all” approach is not feasible across the entire class. While there is a timeline for the second semester to help guide the students (Table 2), much of the scientific discovery is driven directly by the students and their investment in discovering the function of their POI.

**Table 2:** Second‐semester timeline

Week	Module	Assignment type
1	Organize reagents and buffers and finalize protocol for assay	—
2–3	Express, purify, and quantify POI	Revised POI report; chemical order request (reagent inventory)
4–5	Find workable solution/buffer conditions, optimize protein concentration for enzyme kinetics assays, establish controls for these assays	—
6	Group meeting preparation; evaluation of assay; begin determining kinetics parameters (*K*_M_, *k*_cat_, etc.)	Draft of *Materials & Methods* section of final POI report
7–8	Experimental determination of kinetic parameters for each POI	GM presentation 1
9	Present GM 1; begin systematic variation/perturbations of kinetics assays (e.g. substrate variation)	Drafts of *Introduction*, figures, and *Future Directions* sections
10–12	Repeat assays for statistical replicates or troubleshooting; final refinements and kinetics data‐collection	GM presentation 2; poster presentation; final POI report (manuscript format)

### First Semester Modules and Assignments

Modularity and flexibility were major aims of our curricular design, such that the research and/or instructional components of BioLEd can be implemented equally easily at predominantly undergraduate institutions or PhD‐granting research (R1) universities. In other words, we sought to create a course that could be comprehensive, but also amenable to only partial implementation—e.g. at institutions which do not devote a full semester or two to protein biochemistry, or if instructors wish to implement only portions of the curriculum. By creating a modular curriculum, instructors at any institution can choose to incorporate discrete elements of BioLEd into their preexisting courses.

The first‐semester curriculum consists of seven experimental and five computational/discussion‐based modules (Table 1). Students work with their assigned POI for all modules except those involving LDH kinetics (Modules 4, 5). Modules 1a and 2 are designed to introduce students to the literature and online/web resources, and to guide them in finding articles in both the primary and secondary (review) literature that may be relevant to investigating their POI. Using a modification of the C.R.E.A.T.E. method 21, students are guided in reading and critically analyzing research articles related to their POI.

Modules 1b (pipetting) and 3 (buffers) are fairly basic types of laboratory activities, and students are provided with intentionally brief experimental descriptions rather than detailed protocols or specific instructions for a given task. For instance, students might be instructed to “*prepare 100 mL of 1 M Tris pH 8*,” versus “*to prepare a 10x stock of Tris buffer, begin by adding 121.14 g of Tris to a clean beaker and…*.” In our experience, for many students this may be their first experience with stock solutions and careful buffer/solution calculations. Students determine the detailed protocols for making the solutions they need, and they are individually tasked with making any necessary calculations as a pre‐laboratory assignment. This approach helps instill the self‐reliance and proficiency that becomes increasingly important in later stages of the BioLEd curriculum.

Modules 4 and 5 focus on kinetics assays using the enzyme LDH. This activity prepares students for the second‐semester experiments, where they conduct kinetic assays on their own POIs. Module 4 requires students to determine (i) the optimal concentration of LDH enzyme for detecting signal in their spectrophotometric assays, as well as (ii) a suitable range of substrate concentrations for determining LDH kinetics parameters. Students learn how to select proper substrate concentrations to enable determination of Michaelis‐Menten kinetic parameters, including the initial reaction velocity (*v*_0_), maximal velocity (*v*_max_), and the Michaelis constant (*K*_M_). Sometimes, a partner pair discovers that they cannot calculate reliable kinetic parameters because the substrate concentration range initially settled upon did not sufficiently span the hyperbolic *v*_0_ versus [*substrate*] curve. An entire laboratory session is dedicated to processing and analyzing the kinetics data that have been acquired (from raw absorbance measurements, to progress curves, to Michaelis‐Menten plots) and, in some cases, students can repeat the experiment if they realize they did not have an appropriate range of substrate concentrations.

**Figure 4:**
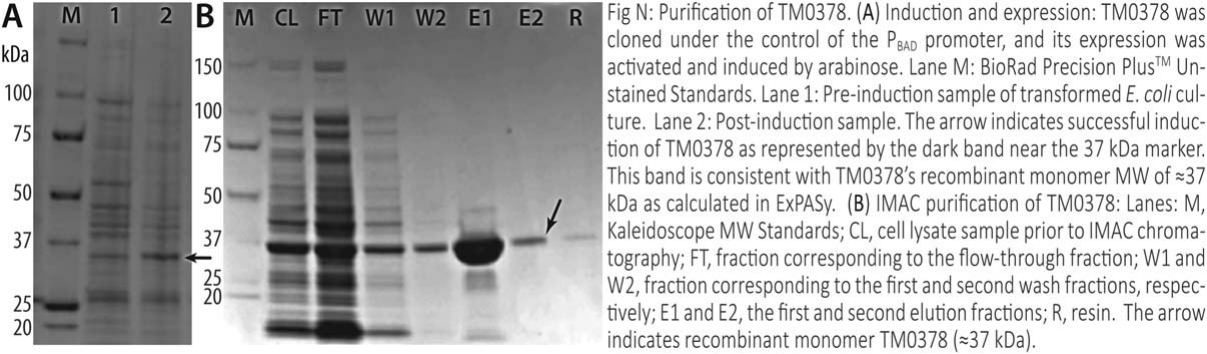
Experimental biochemistry is the core of the BioLEd curriculum. To obtain pure protein samples for kinetics assays, students learn standard techniques of protein induction and over‐expression, cell harvesting/lysis, chromatographic purification, etc., as illustrated by this SDS‐PAGE gel (and associated caption) from one of our laboratory section's POIs.

In Modules 7–9, students learn to transform the DNA plasmid encoding their POI into chemically competent *Escherichia coli*, over‐express the recombinant POI via induction with IPTG or arabinose (depending on the plasmid), harvest and then lyse their *E. coli* cell culture, and finally purify their POI using three types of chromatography (below). These key labs introduce students to the recombinant DNA technology that was used to clone the gene for their POI, as well as the methods used to over‐express and purify proteins both for this and subsequent labs (e.g. second semester). A sample protein expression/purification workflow, as executed by one of our BioLEd student groups, is shown in Fig. 4.

All POIs used in our course were cloned by JCSG into either pBAD‐derived (pMH4) or pSpeedET bacterial expression vectors. These protein constructs feature an N'‐terminal His6× tag, enabling immobilized metal affinity chromatography (IMAC) purification on a Ni^2+^–charged resin. By having students purify their POIs via affinity, gel‐filtration, and ion‐exchange chromatographies, they can both learn these types of chromatography and also conclude—on their own—that, in general, IMAC affords the greatest purity and yield 22. Students also learn electrophoretic protein separation (using SDS‐PAGE) during these modules, and use it extensively in both semesters to monitor their protein expression and purification. Students use these methods to purify their POI in Module 9, and then learn how to quantify samples in Module 10. Students are taught the bases of two common quantitation techniques (dye‐binding and *A*_280_), as well as the caveats of each approach (e.g. the possibility of spuriously high concentrations when using the Coomassie dye‐binding method, if the POI has a disproportionately high fraction of basic residues relative to the calibration standards). Students learn the advantages/disadvantages of each approach, how to execute the technique, and how to analyze the data (standard curves), all while determining the concentration of their POI samples. Module 10 also leverages the dye‐binding quantification method to introduce the concept of protein–ligand binding assays. Ligand‐binding experiments that are tailored to each student's POI are not easily performed because (i) each group works with a unique POI, (ii) the potential ligands to each POI are unknown, and (iii) data for binding isotherms are not readily acquired (at least not with the detection methods and equipment found in most undergraduate biochemistry labs). Rather, the topic of ligand‐binding equilibria is introduced by quantifying the binding of Coomassie to bovine serum albumin, as described by Sohl & Splittgerber 23. Given suitable equipment and available materials, students may propose similar POI–specific experiments in the second semester.

**Figure 5:**
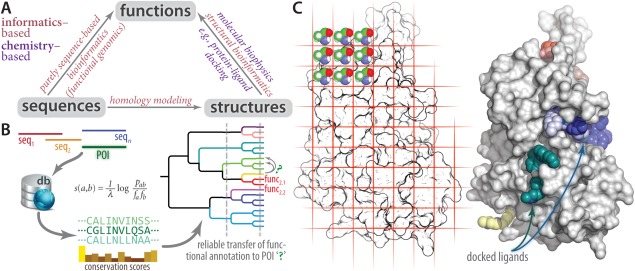
Computational biology is integrated into BioLEd in the context of protein functional annotation. Students learn that both informatics‐based and physicochemical‐based methodologies can be used to investigate the biomolecular sequence/structure/function relationships underlying biochemistry and molecular biology (*A*). For instance, students learn methods such as sequence analysis (*B*) and molecular docking (*C*). Together, these complementary approaches can help elucidate the function of their POI.

The computational biology components (Modules 6 and 11) guide students in using both informatics–based (Module 6) and chemistry–based (Module 11) computational methodologies as a way to quantitatively explore the sequence/function and structure/function relationships for their POI. These modules rely on the deep *sequence* ↔ *structure* ↔ *function* paradigm at the heart of biochemistry (Fig. 5). We introduce students to both families of approaches for inferring protein function: (i) the statistical/data‐driven approach of bioinformatics (Module 6; Fig. 5 *B*) and (ii) the chemical/structure‐based approach, as exemplified by molecular docking (Module 11; Fig. 5 *C*). A key lesson taught here is the *comparative approach* in biology: Students learn that they can use systematic comparisons at the levels of sequence and structure, between their POI and proteins of well‐characterized function, to predict potential functions of their POIs (e.g. substrate specificities). Then, they design experiments to test those predictions in the second semester. Throughout these Modules, students are taught structural bioinformatics concepts and jargon (“homology,” “domain,” “superfamily,” “fold family,” etc. 24), as well as the principles of sequence‐based bioinformatics (e.g. BLAST expectation values). Students learn, for instance, that being able to classify their POI into a particular fold family does not necessarily provide sufficiently detailed information to allow meaningful (*specific* and *testable*) hypotheses for a POI's substrate specificity.

In Module 6, students employ bioinformatic servers, databases, and literature‐search methods to help identify potential enzymatic activities, substrate specificities, and any function‐related motifs in their POI. This is done at the levels of sequence and structure (Fig. 5 *A*). This module demands a highly immersive learning approach and, because this material is new to many students, a more planned approach may be necessary at this stage (e.g. we have had students pattern their workflows after Mazumder & Vasudevan's approach 25 to structure‐guided comparative analysis of protein function). We first introduce basic concepts, including PDB file manipulation 26, sequence alignments, and phylogenetic trees. We then introduce students to powerful bioinformatic tools for (i) structure comparison, both pairwise (e.g. in the Pymol molecular visualization environment 27) and against structural databases (e.g. VAST 28, DALI 29); (ii) integrated structure analysis services (e.g. pdbsum 30); (iii) comprehensive sequence/function databases such as UniProt 31; and (iv) databases and knowledge‐bases with a specific focus on enzyme function (e.g. BRENDA 32) or pathways (e.g. KEGG 33). This Module should emphasize to students that knowledge gleaned from database searches and analyses can be integrated with careful study of any primary literature that might be available for functional characterization of close homologs of their POI.

Module 11 introduces students to what can be learned by detailed analysis of the 3D structure of their POI. Molecular visualization 34, modeling approaches (e.g. homology modeling), and protein/ligand docking 35 form the core of this module (Fig. 5 *C*). Students examine the features of their structure using Pymol, which they are introduced to early in the semester and which we then revisit in class (using, e.g. Supporting Information 2). Students optionally build homology models using Swiss‐Model 36, and conduct docking experiments with PatchDock 37. We recently developed a standalone (non–web‐based) educational workflow for docking that uses the high‐performance AutoDock‐Vina software 38. In this workflow (Supporting Information 3), students learn docking as a powerful *in silico* tool for exploring the ligand‐binding properties (and hence function) of their POI, and the students also learn basic usage of the Linux operating system (this is an exciting first for many students). We have found that students need close guidance in order to learn to distinguish less relevant small molecules in a PDB file (e.g. glycerol from crystallization conditions) from more promising cofactors, metals or other ligands that might be bound, and to learn how to navigate and interpret the vast information content of a PDB file. Similarly, we find that most undergraduates must be carefully introduced to the notion of protein packing in a crystal lattice, and how such packing may relate to the biologically functional oligomeric state; this is an especially important point as regards students' structural analyses of POIs that are suspected to act as multimers.

Another lesson regarding the computational biology modules is that BioLEd is generally the first laboratory course encountered by biochemistry students (at least at UVa) that does not expect specific, preordained, “right‐or‐wrong” answers. Indeed, we have found that a difficulty in facilitating the bioinformatic labs is that many students expect questions to have a single “right” answer; thus, a common pitfall is that many students are tempted to mechanically “plug and chug” data into bioinformatic servers, rather than explore, critically analyze, and ruminate about the results for their POI. Instructors and TAs can preempt this difficulty by repeatedly emphasizing that *active investigation* and *digging* (*data mining*) will yield interesting discoveries and putative leads about possible POI functions. During all computational biology sessions, the instructional staff should engage the students about their findings in “real time.” For instance, as students are conducting sequence similarity searches for homologs, TAs can question them about the total number of “hits” detected beyond the statistical threshold, how the number of to *new* hits changes after 1, 5, 10, … iterations of PSI‐BLAST 39, 40, and so on.

### Second Semester: Summary

Unlike the first‐semester biochemistry laboratory, a pre‐set syllabus of laboratory modules and associated protocols/guides is not provided to students in the second semester. Instead, the students are charged with planning their work: They formulate a strategy and timeline in consultation with the instructors. We provide a general outline of experimental progress, as an idealized plan for students to follow (Table 2), but they are free to propose deviations from it. As with “real” biochemical research, students often find that they must adapt their second‐semester plans based on the outcomes of their individual experiments and the general behavior (solubility, etc.) of their POI.

Students over‐express and purify their POI using the knowledge they gained in the first semester—namely, the chromatographic purification method that gave the best results with their POI. (In general, most students proceed via IMAC with their (His)_6_–tagged POIs.) Next, the purified POI has to be exchanged into a buffer in which the protein is soluble at their working concentrations, and which is compatible with the planned enzymatic assays. The students must discover what types of buffer conditions others have used to study homologous proteins, and what conditions work with those homologs that have been confirmed as having the same enzymatic activity that the POI is thought to have. Designing this experiment requires students to use the literature skills that they developed in the first semester.

Determining a suitable buffer, both for enzyme storage and enzyme assays, can be challenging and time‐consuming. General guidelines, including a discussion of the importance of salts, ionic strength, pH and protein concentration, help the students get started in selecting buffers, and also provides a starting point for troubleshooting solubility issues; nevertheless, suitable buffers typically must be determined by empirical trial‐and‐error. Because each POI has already successfully traversed the typical structural genomics *cloning* → *over‐expression* → *purification* → *crystallization* pipeline, in principle students should be able to obtain high yields of pure, soluble protein for each POI target. Regardless, roughly one‐quarter of our POIs over the past few years have proven exceptionally challenging, and simply obtaining conditions which allow the protein to remain soluble might be judged as being sufficient (in terms of student grades).

After obtaining pure protein in a suitable buffer, students must optimize the POI concentration that will be used in enzymatic assays throughout the semester. We define an “optimal” amount of POI as enough protein to obtain a reliable kinetics signal, but as little protein as possible so that many assays can be performed with one preparation; in addition to maximizing throughput, this strategy reduces the variation between sample preparations. Optimizing the POI concentration requires certain concepts to be understood. The spectrophotometer “blank” and the “negative control” (for background rate subtraction) are especially confusing to students, partly because there is not a standard/well‐defined terminology in the literature. The blank for the spectrophotometer can be confused with an “enzyme blank,” which actually is more accurately considered a “negative control,” “blank rate,” or “background rate.” For example, for the LDH assays we teach students that the spectrophotometer *blank* consists of all assay solution components except for the enzyme and any light‐absorbing cofactors being monitored (NADH); the *blank* is used to set the absorbance of the spectrophotometer to zero. The *background rate* is the change in absorbance signal of the full assay solution—minus enzyme—over the same length of time that enzymatic activity is monitored. Establishing the background rate is important because the next step is to discern a *significant* signal versus background noise. Concepts such as the instrument's detection limit and the background signal must be thoroughly discussed in order to ensure that students can discern when their data reveal authentic activity, as opposed to data that differ only insignificantly from the background rate.

Some target POIs are almost certainly misannotated 41 or annotated at only low functional resolution in public databases. This means that the substrate(s) the students chose to test might be inappropriate for the POI, yielding negative results. Distinguishing true negative results from student error requires a positive control. However, a true positive control is impossible because the POI functions are unknown. When coupled reactions 42 are used, we provide students with the substrate of the coupling enzyme, allowing them to observe and measure the activity of the coupling enzyme alone. Finding activity for the coupling enzyme(s) alone reassures students that their reaction conditions are favorable for the planned assay. In the case of a direct assay, a commercial enzyme (if available) is used as a positive control, again providing assurance that assay conditions are compatible with enzymatic activity. In addition to planning suitable controls, students should plan to test alternative substrates in the event that their putative function is not supported; selection of viable alternative substrates can be guided by bioinformatics, docking results, and the literature.

Upon initial detection of activity and optimization of POI concentration, students determine the kinetic parameters with one substrate. Doing the experiment in triplicate to obtain standard deviations is important—students are typically intrigued by the variation they find, and they become more critical of articles in the primary literature that do not report standard deviations or other statistical estimates of error. Once students have acquired and processed the kinetic data, they are encouraged to systematically vary the assay to begin investigating the catalytic mechanism, protein stability, and/or substrate specificity. Students often choose to vary the pH, temperature, available metal cofactors, or to test the effects of inhibitors that they chose based on bioinformatic analyses.

### Second Semester Assignments

This research‐based curriculum involves assignments that are atypical for a standard laboratory course. For the first assignment of the second semester, student teams prepare lists of required chemicals and an outline of their planned experimental (kinetic) assays. Next, independently written assignments require each student to detail the materials and methods used in their work (week 6), and to write a report with introduction, figures, and future goals (week 9). Both of these assignments prepare students to write a POI manuscript that is due at the end of the semester (week 13). Students benefit by having the final, large‐scale assignment consist of these sub‐tasks distributed throughout the semester, and they are also able to incorporate the feedback they receive on the smaller assignments into the final manuscript. Having these assignments earlier in the semester also ensures that students are sufficiently immersed in their POI. In addition to the final manuscript, each POI group (Fig. 3) creates a poster for an end‐of‐term poster session, simulating the experience at a scientific conference; while the poster is prepared as a group effort, individual students take turns presenting the poster to the instructors and teaching assistants. In the past few years, dozens of BioLEd students have presented their results as posters at a local meeting of the American Chemical Society; this late‐April event is opportunely timed just before the end of each Spring term, and similar regional events likely can be found near other institutions considering a BioLEd‐based curriculum.

Two group meetings (GM) per POI are held in the second semester, as detailed in the *Teaching Communication & Critical Thinking* section (below). These meetings mimic GMs held in research labs, our aim being to encourage interactions among students and between students/instructors, and to train students in effective scientific communication. To help students prepare for their final poster and manuscript, instructors should provide discussion and feedback on student figure preparation, how data are presented (types of plots, etc.), and the overall quality of the GM presentation. Instructors also analyze and discuss the scientific content of these presentations, so as to fully grasp the data that students are generating as well as the overall progress of each POI project. The GMs are spaced roughly ⅓ and ⅔ of the way through the semester, giving milestones to help students remain focused on the ultimate goal of characterizing the enzyme kinetics of their POI.

### Second Semester Grading

Group‐based projects, which are at the level of an entire POI group rather than individual students or partner‐pairs (Fig. 3), comprise a relatively large share (≈30%) of a student's final grade in the second semester. This group work includes making GM presentation slides, collaborating on the end‐of‐term poster, and writing the final POI manuscript. Non‐group components of the second‐semester grade include an individual student's performance on the GM presentations (≈15%); his weekly quizzes, notebook, and effort grades (≈8%, 7%, 10%, respectively); and other individual laboratory reports during the term (≈30%).

Because the BioLEd curriculum is one of real research, the grades for the final project and presentations are based not on “positive” results, but rather on criteria such as students' use of the scientific method (e.g. systematic controls), scientific inquisitiveness, problem‐solving efforts, resourcefulness, and overall effort. Some of our past POI targets have been difficult *in vitro*, generally due to protein solubility issues or because no enzymatic activity was detected. In such cases, little to no kinetics data were obtained by students. A lack of “results” from such POI groups does not ensure a low grade; instead, it is made clear to students that they would be expected to (i) use the primary literature to investigate potential reasons for difficulties, (ii) develop troubleshooting scenarios, and (iii) rely more heavily on computational biology to investigate the putative function of their POI. In this way, students learn that they are engaged in very real scientific research; we have found that many students embrace these challenges.

## Infrastructure

As implemented at UVa, the BioLEd‐based course meets for a one‐hour class and four‐hour laboratory session per week. On occasion, less formal review sessions or office‐hours are also offered (e.g. a session dedicated to the Michaelis‐Menten and related kinetics equations). Two research‐active faculty, one full‐time instructional laboratory support specialist, and six graduate student TAs serve the third‐ and fourth‐year undergraduates enrolled in the year‐long course. Notably, the BioLED approach scales well: Of our 100+ chemistry graduates per year, typically ≈80–90 have enrolled in our biochemistry laboratory (predominantly chemistry majors with a biochemistry focus). There are six laboratory sections per week, each led by one graduate TA. Each TA/laboratory section is assigned two POIs; each POI typically has five to nine students, working in pairs or triples (Fig. 3). In total, 12 POIs are studied each year, distributed across the six laboratory sections.

Laboratory sections meet in one of two laboratory spaces. Each laboratory is equipped with, for every two to three students, a UV/Vis spectrophotometer, a gel electrophoresis setup, and a stir plate. Each laboratory also has shaker‐incubators for cell growth and protein expression, a standard centrifuge, pH meter, scales, assorted chromatography columns, and other typical biochemistry laboratory equipment.

### Teaching Assistants

The course described above requires six teaching assistants; thus far, we have accommodated student:TA ratios as high as 18:1. TA preparation is vital to the success of this laboratory. Most graduate students in the department teach in their first two years. Because most of the TAs are new to research themselves, several hours are scheduled to train them before the Fall term begins. TAs are introduced to the pedagogical principles, best practices, and instructional strategies underlying the BioLEd curriculum. During the semester, TAs are expected to perform all of the computational labs and create the keys used in grading those assignments. Each laboratory protocol is discussed in detail (at a TA meeting near the start of each week) in order to identify thin areas in a TA's knowledge‐base. Also, novice TAs who teach a Wednesday or Thursday section are encouraged to observe at least part of a laboratory earlier in the week. Because we find that TAs often hesitate to reveal when something is new or unfamiliar to them, the TA training module is evolving to include an actual dry‐run of each laboratory technique (rather than simply a discussion).

## Content Delivery, Active Learning

The limited time for instructor–student interaction is a difficulty in implementing the BioLEd curriculum in a typical (3‐credit) undergraduate biochemistry laboratory. In one hour of lecture per week, the instructor may seek to cover the theory of the method(s) being used in laboratory that week, practical aspects of implementing a method, various aspects of statistical data analysis/interpretation, and so on. All the necessary content cannot be covered in a one‐hour lecture. In addition, data analysis/interpretation is more effectively learned actively, rather than by lecture. Thus, the typical lecture has been replaced with an inverted lecture style 43, 44, 45. Lecture content was recorded as brief (<15 minutes) slideshow videos and supplemented with reading assignments. Practical execution of laboratory methods was also provided as videos, either found online or created in‐house. The weekly lecture hour was thereby freed so the instructor could actively work through sample calculations, describe anticipated data/graphs, interpret data, demonstrate the usage of software and databases, and answer any troubleshooting questions.

Interactive teaching 43, 46, 47, 48, 49, 50, 51 is an effective tool for delivering most of the laboratory course content. For instance, for the lecture on ion‐exchange chromatography, students are asked to draw a putative chromatogram on the board. One student volunteer might draw the axes (*A*_280_ and ionic strength as *y*‐axes), while others may make changes based on feedback from the class and instructor. Further student volunteers will then draw a typical *A*_280_ trace and ionic‐strength trace. A final student might then be asked to sketch the expected SDS‐PAGE gel of specific fractions from the chromatogram; this is an especially valuable exercise for gel‐filtration chromatography, where any oligomeric POI that elutes should migrate at the mass of a monomer on a denaturing gel. The class is encouraged to add or otherwise edit what is drawn on the chalkboard, and especially to ask questions. This interactive format engages students and encourages active participation. Those students not actively participating at any given moment are nevertheless thinking about what they would draw, and are able to work through their ideas via discussion. During some lecture times, the class works through problems in pairs or small groups; representatives from each group volunteer to share their answers. This format is particularly helpful for the Buffers & Solutions module (Module 3, Supporting Information 1), as the concepts of stock solutions and dilutions are cemented via calculations and the practice of making the solutions.

Another active learning strategy—concept mapping—is introduced in week two (Module 2). First, the instructor shares a concept map about a topic that should be familiar to students from past coursework (e.g. hemoglobin). The instructor explains how the hemoglobin map was created, and that each concept map is unique. Another familiar topic is then chosen, and individual students begin creating a concept map on the chalkboard, connecting ideas, facts and concepts related to the new topic. The relationship between the concept map and literature search keywords is easily introduced by having students combine words from the map, use these as literature search queries, and then compare the results.

Active learning can also be used to teach data analysis. A data figure can be projected, and the students can be asked questions that are either factual (e.g. *what method was used to generate the data? what controls are present/missing?*) or interpretative (e.g. *what hypothesis might these data address? what conclusions can be drawn?*). For each type of question, multiple answers are heard, compared, and discussed amongst the students and instructors (including TAs). This instructional mode is especially important in the second semester, when some student groups start generating potentially large amounts of kinetics data for their POIs. In the second semester, those lecture hours that are not scheduled for activities such as GMs can be used to reinforce important concepts (e.g. analyzing progress curves to extract Michaelis‐Menten kinetic parameters), address any recurring troubleshooting issues, and so on.

All of these active and interactive learning methods have been highly effective in the BioLEd curriculum, based on our initial assessment results (described below). In general, the instructional tools and best‐practices to be deployed in a specific course will vary with the exact concepts, sets of students, and instructors involved; this aspect of curriculum design should be researched by an instructor to identify what are likely to be the most suitable styles for a given course 44. Numerous active learning options exist for teaching different types of concepts (e.g. 44 and 52).

## Target Selection & Preparation

The proteins selected for students as target POIs generally meet certain criteria: (i) a 3D structure of the protein is available; (ii) the protein function is unknown/unreported; (iii) the putative function is likely enzymatic (as inferred from bioinformatics); (iv) the enzymatic reaction can be monitored via spectrophotometric assays (either directly or via coupling reactions); and (v) all substrates, cofactors, and coupling enzymes are commercially available and are affordable. Before the term begins, a PhD‐level instructor evaluates each POI candidate against these criteria. As targets are selected, corresponding clones are requested from collaborators at the JCSG or are purchased from Arizona State University's *Plasmid Repository* (http://dnasu.asu.edu/DNASU). To verify that correct target plasmids have been obtained, and to prepare materials for the students for Module 7, plasmid DNAs are mini‐prepped/sequenced by the instructional staff before the term begins.

Based on our experiences with over 40 POI targets, we recommend avoiding dehydrogenases with vague annotations (e.g. an “alcohol dehydrogenase”) unless the operon structure or other bioinformatic data strongly suggest a specific substrate. As an example, we have had a student group who surveyed over 20 substrates for one POI with no positive results, implying that “dehydrogenase” is an insufficiently precise descriptor for this class of enzymes. Though negative outcomes do not affect the students' course grades (see above), confidence and morale can become diminished in these POI groups.

## Facilitating Group Work

Along with the call for science to be taught in a more experiential manner, there has been a call for teaching in a more collaborative and cooperative way 53: “*The collaborative nature of scientific and technological work should be strongly reinforced by frequent group activity in the classroom. Scientists and engineers work mostly in groups… Similarly, students should gain experiences sharing responsibility for learning with each other*.” In addition to learning the skills of working within a group, students often learn and retain more when they work in small groups on projects (e.g. cooperative learning 54, 55) versus other instructional formats 54, 55, 56, 57. BioLEd students experience cooperative learning, the characteristics of which include (i) students working in small groups, (ii) students experiencing shared learning goals (and tasks that may differ from those of other groups), and (iii) grades that are based on both individual work and group work.

Group work can be difficult to implement, largely because of the personality and aptitude differences inherent to any collection of human beings. More than three years of experience in implementing the BioLEd curriculum reveals that many challenges directly stem from intra‐group dynamics. Common issues include (i) a student feels that the workload/contributions in their group are unequal; (ii) a lack of communication, electronically and in person; and (iii) irresponsibility on the part of one group member hampers the entire group (e.g. someone forgets to come into laboratory to start an overnight culture, thus delaying their entire group by at least a day). These types of issues are common to cooperative learning, and can be addressed by incorporating the following practices:

(i) *positive interdependence*: students learn that their success is tightly coupled to the contributions and success of others in the group
(ii) *face‐to‐face positive interaction*: students must be encouraged to directly interact, both during discussions (such as the GMs) and by sharing information
(iii) *individual and group accountability*: students are held accountable both for their individual work and for contributing sufficiently to the group project; thus, both individual and group grades factor into the overall grade
(iv) *group processing*: students are given opportunities to “grade” their group's functionality, and to discuss what have been positive and negative aspects of their experience working in their POI group

In the first semester, students have a strong incentive (individual grades) to stay on‐task and be prepared each week (quizzes, pre‐laboratories). This intentional course structure helps reinforce student independence. In contrast, the second semester leaves preparedness and time management to the students. Also, grading methods are required that specifically address the issues associated with group work. A balance of group and individual grades was found to be crucial in order for students to appreciate that their grade does incorporate their personal effort and intellect, regardless of the effort and performance level of their peers. For example, the overall grade for the second‐semester GM presentations includes group and individual subtotals. Effort reports are prepared by each student and turned‐in with select assignments (Supporting Information 4 is a sample). These reports are vital for an instructor's evaluation of the group, and also for students to pause and consider the contributions of each group member. Though students tend to be generous with one another in scoring overall effort, students who do not contribute are easily identified by this mechanism. Questions pertaining to what each individual student brought to the group, and what the student learned from the group, help students appreciate the benefits of working together cohesively.

## Teaching Communication & Critical Thinking

### Group Meetings

GMs occur twice in the second semester, at weeks 7 and 10. Each GM is attended by the instructors and TAs for that POI. The meeting format mimics that in most research labs. Students present a collaboratively prepared slide presentation in a small group setting; presentations are followed by discussions. In advance, students are given an outline of what sort of information should be included in their presentation slides. To ensure that each person is familiar with all of their group's work, students are told to be prepared to present any segment of the presentation; slides are assigned to individual students at the start of the GM. These meetings are kept intentionally informal and interactive, and it is useful to bear in mind that many undergraduates will be nervous about speaking in front of their professors.

The GMs are valuable on many levels. First, science majors are rarely expected to present their work in class, and therefore they do not gain experience in articulating and defending their ideas “on their feet.” The BioLEd curriculum affords opportunities for students to gain confidence in communicating their work via scientific/technical speaking, in a calm and welcoming environment. Second, the GMs help instructors track the students' progress with each POI, individually and as a group. In classes with large numbers of students and sections, instructors likely will be unable to stay abreast of each POI project without such meetings. The GMs also allow for interactive brainstorming and troubleshooting. Because much of this course entails group work, a student's individual turn in presenting part of the GM is a key opportunity to demonstrate her mastery and ownership of the work (i.e. apart from the group work to prepare the slides); also, the instructors can gain a sense of how the group is functioning. An important result of the GMs is enhanced faculty‐student interactions in an intimate setting. Studies indicate that such environments are especially important for novice students, whose needs differ from students with research experience 58, 59, 60. Personal interactions with research mentors can address some of the differences, by providing guidelines and orientation, as well as socialization in the traits of scientific researchers.

### Final Manuscript

The BioLEd students' research culminates in a manuscript prepared in the style of a scientific publication. Students work towards this final report throughout the semester in discrete stages, corresponding to the sections of a typical scientific article (*Introduction*, *Methods*, etc.). For the final report assignment, students merge their adapted *Materials & Methods* (from week 6) and *Introduction*, together with figures and future work (from week 9) and newly written *Abstract*, *Discussion*, and *Results* sections. Much of this final paper involves bioinformatics, which students were introduced to in the first semester and urged to revisit since then. For instance, the *Introduction* contains the students' hypothesis about the function of their POI (e.g. substrate specificity of a putative aminotransferases), which forms the starting point for the second semester's group work. Students learn that their hypotheses have to be justified, largely via bioinformatic results with their POI and by analysis of the salient literature for any homologs.

The manuscript is a group project. There are many reasons for this. First, there are several facets to the manuscript, and each student brings different strengths to bear on the research and writing; this reflects how research groups actually work in academia, national labs, industry, etc. Second, students work throughout the semester to study a single POI in groups of up to six to eight students (Fig. 3). Early on, we prod students to consider working on different aspects of the POI (i.e. as a synergistic group rather than just a collection of individuals); this way, they accomplish more than they thought possible. However, only one manuscript per POI is accepted. Thus, students are made to work together to craft their findings into a cohesive description. Achieving this goal teaches students efficient scientific communication. Finally, because peer review and critical data analysis are important skills for scientists, groups are encouraged to hash through a series of drafts and edits. We also require a breakdown of each individual's contribution to the manuscript; this accountability helps promote a fair division of labor towards the manuscript. The final manuscript may develop into a line of further work: If the final results for a given POI are definitive, demonstrating either (i) the annotated enzyme activity, (ii) absence of the predicted activity/substrate specificity or (iii) some other activity/specificity, then the instructor can work with any interested students from the POI group to draft a new annotation entry for submission to TOPSAN (see above). And, if the POI results are publishable, then instructors can recruit students from the POI group towards such efforts. At least some further experiments (beyond those in the final project report) are generally required before being able to publish, and such work can be pursued the next Summer or academic year (e.g. for research credit); indeed, one recent student developed his BioLEd project into an MSc thesis in our own (research) laboratories.

### Poster Preparation and Presentation

Most undergraduates are unfamiliar with the ways scientists present and share their work at meetings. Preparing a scientific poster requires students to mine their data and results (which are reported in detail in the final manuscript), distilling the work into only the most compelling results and effective figures. In addition, students must be prepared to lead an audience through the contents of their poster. Students who do this well typically possess a deep knowledge of both their POI as well as each group members' contributions towards characterizing the POI. The poster exercise gauges student familiarity with *what* work was done as well as their grasp of *why* particular sets of experiments were pursued.

The poster presentation is also an opportunity for students to practice scientific speaking. Unlike the GMs, each student walks the instructors and TAs through the poster and explains the entire project on their own. So, while the poster is generated as a group effort, the posters are presented individually. Some students who do not do well on written work are found to shine during oral presentation, with their depth of knowledge more readily apparent in these real‐time interactions. Thus, student performance is optimally assessed by not limiting the graded work to only written assignments.

## Assessment

Our approach to course assessment was multi‐pronged, the overall goal being to gauge the effectiveness of the new BioLEd curriculum. We evaluated the curriculum in three ways: (i) student gains in scientific content were assessed by us via pre–/post–course tests; (ii) student performance and content gain were self‐assessed with pre–/post–course surveys; and (iii) university‐wide course evaluations were used. (All assessment activities were approved by the UVa Institutional Review Board for the Social & Behavioral Sciences [#2010041200] and were in compliance with their policies.) We also surveyed past students on their opinions of the course; specifically, we questioned BioLEd alumni on whether this course gave them a deeper understanding of biochemistry and enabled them to approach scientific problems more effectively.

### Assessment of Student Performance on Assignments

For purposes of both grading and course assessment, we defined the four learning gains shown in Fig. 1: (i) Aims & Concepts, (ii) Experimental Design, (iii) Data Processing, and (iv) Broader Context. These four learning gains are further defined by eight focal areas: (i) laboratory skills, (ii) broad biochemical knowledge, (iii) reading/comprehending scientific articles, (iv) written and oral communication, (v) group dynamics skills, (vi) investigative skills, (vii) critical thinking, and (viii) problem‐solving skills. Each learning gain and focal area can be evaluated by specific outcomes (examples are given in Fig. 1). Outlining learning gains—and using detailed grading rubrics based on these gains and focal areas—are important steps in assessing student performance in a newly developed curriculum. In the first semester, the TAs grade assignments using rubrics that we developed for two purposes: to enable assessment of students in our four learning gains, and to help focus a TA's grading efforts on those concepts specific to each assignment (a sample rubric is shown in Supporting Information 5). Though necessarily detailed, BioLEd rubrics cannot be *too* specific because each report may be quite unique (reflecting the properties that are unique to each POI).

By having TAs complete rubrics when grading student assignments, the scores become more reliable and consistent for students within one section and also among different sections (different POIs, different TAs). When graded assignments are returned, students can see what they did well and what areas might require improvement. Furthermore, because the rubrics are based on our learning gains, TAs and instructors can refer to the rubrics for the main focus of a given assignment. If many students seem to struggle on particular assignments, then TAs/instructors can begin to detect patterns, such as a particular learning gain that may require more attention in the classroom or laboratory. In addition to assessing student performance via well‐defined assignments, we also used concept inventory tests to assess content gain and retention at the start of the first term, end of the first term, and end of the second term. The initial results of these studies (outlined below) indicate that most students in the BioLEd curriculum demonstrate sustained learning gains in almost all topics, across the entire year.

### Student Self–assessment of Learning

An assessment mechanism using pre‐ and post‐course surveys, created with the web‐based *Student Assessment of their Learning Gains* (SALG) program 61, was used to examine student confidence levels and self‐reported learning gains. The surveys use five‐point Likert–scale questions, wherein students self‐rated their understanding, skills, and attitudes for various topics that were covered in the course (Supporting Information 6 provides sample questions). Answers ranged from “A Great Deal” to “Not at All,” with a “Not Applicable” option also available. To facilitate calculation of scores, possible answers were given numerical values as follows: “Not Applicable” = 1, “Not at All” = 2, “Just a Little” = 3, “Somewhat” = 4, “A Lot” = 5, and “A Great Deal” = 6. The surveys also include free‐response questions, enabling participants to offer suggestions for course improvement.

**Figure 6:**
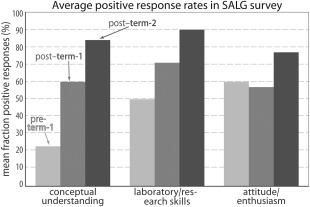
SALG surveys reveal positive response rates for three criteria: understanding of biochemical concepts (left), laboratory/research skills (middle), and attitude/enthusiasm for biochemical research (right). In each triad, representative data are shown for pre–term‐1 (light grey), post–term‐1 (medium gray), and post–term‐2 (dark gray); in a year‐long course, these terms correspond to the start of Fall semester, end of Fall, and end of Spring, respectively. Numerical values and further details are discussed in the text.

A chief goal of our assessments was to determine if students were learning—and felt that they were learning—the concepts we hoped to teach. As a representative example, we gave surveys at the start of the Fall 2011 term, at the end of Fall 2011, and at the end of the Spring 2012 term; these results are denoted “pre‐term‐1,” “post‐term‐1,” and “post‐term‐2,” respectively, and are shown in Fig. 6. (There are generally no new students in our Spring terms, as completion of the Fall course is required, so a “pre‐term‐2” pre‐survey is unnecessary.) In the pre‐term‐1 survey, students rated their understanding of the conceptual topics we covered at a mean value of 3.62 (SD = 1.16); the average was 4.78 (SD = 0.84) in the post‐term‐1 survey, and 5.22 (SD = 0.76) in the post‐term‐2 survey. When asked about their understanding of the presented topics in pre‐term‐1, only 23% of the students answered positively (Fig. 6, left‐most bar), while the means rose to 60% for post‐term‐1 and 84% for the post‐term‐2 survey. This represents an increase of 61% from the start of the course (i.e. pre‐term‐1). The students self‐assessed their laboratory/research skills at a mean of 4.48 (SD = 1.00) in the pre‐term‐1 survey, 5.00 (SD = 0.77) in the post‐term‐1 survey and 5.33 (SD = 0.68) in the post‐term‐2 survey. Half of the students rated their laboratory skills positively (“A Lot” or “A Great Deal”) in the pre‐term‐1 survey, 71% in post‐term‐1, and 90% in the post‐term‐2 survey, giving an increase of 40% from the start of the course (Fig. 6, middle bars). With respect to their attitudes and enthusiasm for the subject of biochemistry (Fig. 6, right bars), students reported an average of 4.65 (SD = 1.06) in pre‐term‐1; 4.73 (SD = 1.02) in the post‐term‐1 survey and 5.00 (SD = 0.95) in the post‐term‐2 survey. Sixty percent of students reported positive attitudes in pre‐term‐1, 57% in post‐term‐1, and 77% in post‐term‐2 (an increase of 17% from pre‐term‐1); though there was an increase in the averages for this category, the magnitude was significantly less than in the other two categories.

These SALG data can be separated into the learning gain categories used in our rubrics (i.e. *Aims & Concepts*, *Experimental Design*, *Data Processing*, and *Broader Context*). The SALG data reveal that students rate their abilities in *Aims & Concepts* increasingly positively throughout the course: 28% in pre‐term‐1, 64% in post‐term‐1, and 91% in the post‐term‐2 surveys. In the *Experimental Design* category, 26% of students reported positive ratings in the pre‐term‐1 survey, 41% in the post‐term‐1, and 85% in the post‐term‐2 surveys. Similarly, *Data Processing* demonstrated an upward trend, with students self‐reporting positive ratings of 29%, 77%, and 93% in pre‐term‐1, post‐term‐1, and post‐term‐2, respectively. Finally, 27% of students positively rated their grasp of *Broader Context* aim in the pre‐term‐1 survey, 53% in the post‐term‐1, and 83% in the post‐term‐2. Overall, the fraction of students who consider BioLEd as having improved their skills and knowledge in biochemistry increased throughout the year‐long course.

### Student Experiences: A Retrospective Survey

A major aim in BioLEd's development and implementation has been to teach undergraduate biochemistry majors how to conduct scientific research in a realistic setting. Were this achieved, a direct consequence should be a sustained increase in student confidence levels in their scientific knowledge and abilities, as well as a positive overall experience. A post‐course survey was created (using QuestionPro) and was emailed to students who had completed both semesters of BioLEd; sample survey questions are given in Supporting Information 7. The survey was conducted anonymously, and a monetary lottery was used to incentivize participation. The survey largely used four‐point Likert–scale questions, ranging from “Strongly Agree” to “Strongly Disagree,” and also included free‐response questions.

**Figure 7:**
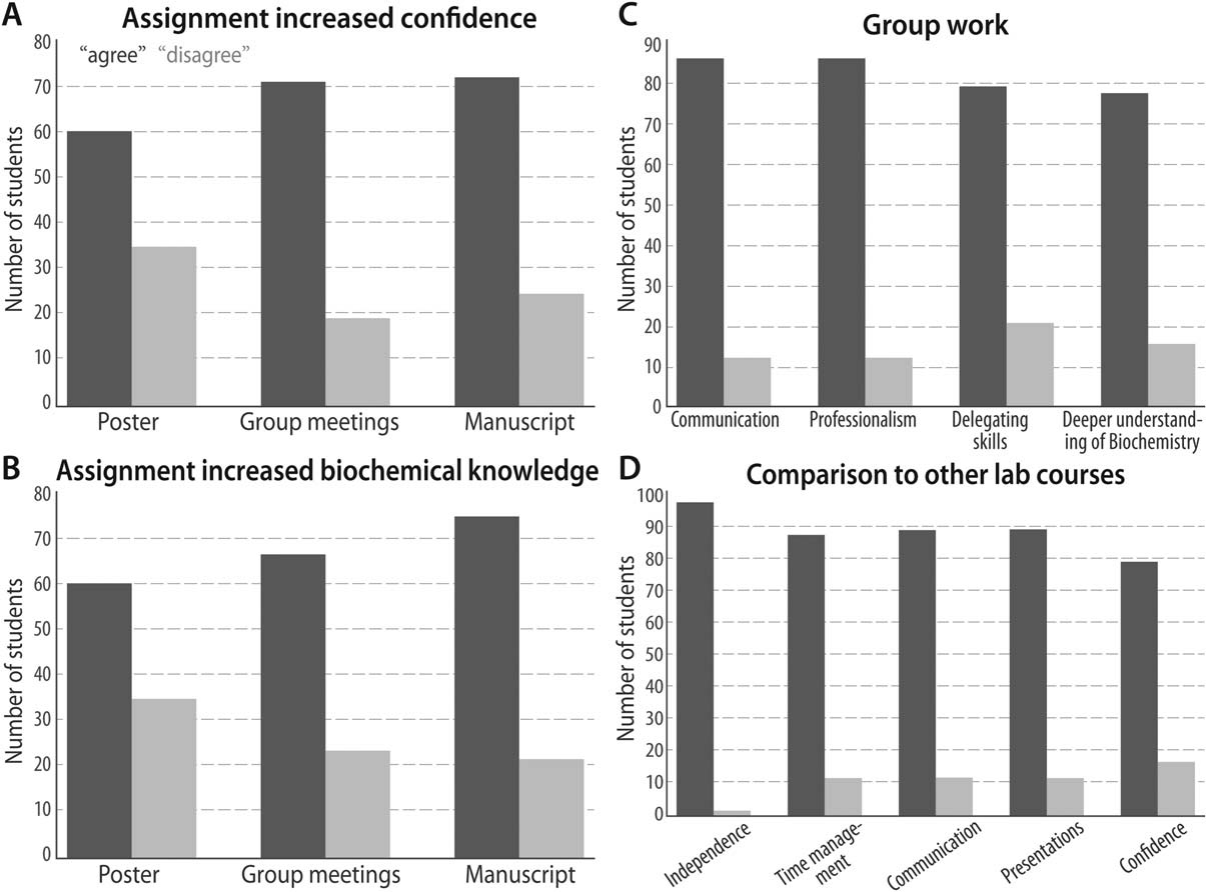
Retrospective surveys of recent BioLEd students show improvements in student scientific confidence (*A*), biochemical knowledge (*B*), and ability to work in a group (*C*); the curriculum also compares favorably to other laboratory courses taken by the students, based on the criteria listed in (*D*). Numerical details and further discussion are in the text.

Of the 128 students initially contacted, 56 completed the survey. Of these, 92% reported that they had earned a cumulative grade of “B” or better in the two semesters; this is consistent with the typical average course grade over two semesters (an 84.6%, SD = 6.75). Though some participants did not complete the entire survey, those portions that were completed were factored into the statistics for individual questions; surveys with an incomplete state were assumed to be due to testing fatigue rather than inaccurate answers. These retrospective surveys are summarized in Fig. 7, and some of the findings are described in the remainder of this section.

The survey primarily aimed to address two questions: (i) did the course (or specific parts of the course) increase student confidence in their research, and (ii) did students feel that the course (or specific parts of the course) provided a deeper knowledge of biochemistry? Sixty percent of the students reported that they “Agree” or “Strongly Agree” that poster presentations gave them more confidence in their research and gave them a deeper understanding of biochemistry (Fig. 7 *A*). Seventy‐two percent reported an increase in overall biochemical confidence, and 75% attested to a deeper understanding of biochemistry as a result of their collaborative manuscript writing (Figs. 7 *A* and 7 *B*). Similarly, 71% of participants reported that the GMs gave them constructive feedback to improve their research, and 67% felt that they had a deeper understanding of biochemistry because of these GM presentations (Figs. 7 *A* and 7 *B*).

Many of the above aspects of the course evaluation reflect *group work*, which measures the ability of an individual to cohesively work together with others to generate a final product. Though group work can be difficult for students to manage, many reported it as a positive experience (Fig. 7 *C*): 85% of students testified to learning how to better communicate with their group members and to work with them professionally, and 78% of the participants reported that they learned how to better delegate tasks within their group. Overall, 76% reported a deeper understanding of biochemistry because of the group work inherent to BioLEd (Fig. 7 *C*, right‐most bars).

Recent student participants were also asked to rate the BioLEd‐based course in relation to other laboratory courses that they had taken (Fig. 7 *D*). Students overwhelmingly “Strongly Agreed” or “Agreed” that the BioLEd course (i) encouraged more independent thinking (97%); (ii) taught better time‐management skills (87%); (iii) taught more effective scientific communication skills (88%); (iv) better prepared them to present scientific information (88%); and (v) encouraged greater confidence in their scientific knowledge (78%), versus other laboratory courses completed during their undergraduate studies.

The above results substantiate BioLEd's goals, design, and implementation, at least in terms of the confidence levels and deeper understanding of biochemistry that students can achieve by being taught via activities that typify research environments—GMs, compiling research results into manuscripts, collaborating on poster presentations, and so on. These elements of the BioLEd curriculum appear to be vital in developing the communication and critical thinking skills necessary in science. The survey that we administered was rather thorough in order to allow detailed assessment of student learning and hints for future course refinements. The level of detail, however, possibly resulted in testing fatigue; also, a four‐point Likert system can be too coarse (e.g. how “strongly” a participant may agree/disagree with a statement varies somewhat, and is not readily controlled for). Future assessment efforts may consider finer (5‐ or 6‐point) scales, and perhaps dividing the one monolithic survey into two discrete components.

### Assessing Content Gain via Pre‐ and Post‐course Tests: A Vignette

**Figure 8:**
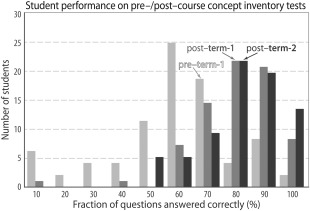
Pre– and post–course concept inventory tests were used to assess student learning and retention of scientific content. This histogram plots data for pre–term‐1 (light grey), post–term‐1 (medium gray), and post–term‐2 (dark gray); numerical values and further details are presented in the text.

A 20‐question “concept inventory” test was administered at the start of the first term, at the end of the first term, and at the end of the second term. These time‐points in a year‐long curriculum are labeled pre‐term‐1, post‐term‐1, and post‐term‐2, respectively (as above for Fig. 6). As incentive, students received five points extra‐credit for completing these quizzes. The questions were designed to address our learning gains (described above), and varied in complexity from highly practical (e.g. read the volume delivered from a pipette image) to the higher‐level skills required to critically interpret kinetics data results; a control question was used that concerned material not included in the course. As shown in Fig. 8, students demonstrated substantial learning gains over the year‐long course. The class mean improved from 52% to 77% to 79% (Fig. 8) of the questions being answered correctly, with a concomitant decrease in the standard deviation (4.49, 3.02 and 2.65 for pre‐term‐1, post‐term‐1 and post‐term‐2, respectively).

### Summary of Assessment Findings

Our initial assessment and evaluation of BioLEd indicates that this inquiry‐driven curriculum provides a sound education in biochemical research, and that student learning is sustained throughout a full year. Students excel in each learning gain over time, as measured both objectively (pre‐/post‐course tests) and more subjectively (SALG results). In addition to the assessments, feedback and anecdotal comments via the UVa course evaluation system have led to many curricular improvements. Past students have recognized the benefits of this type of curriculum, having rated the BioLEd course as more beneficial than any other laboratory course they have taken. Future work could include identifying sets of comparison groups for more thorough and systematic assessments of the BioLEd curriculum; for instance, control groups could be utilized, both at other institutions and as implemented at UVa (e.g. in a parallel laboratory section taught using a more traditional format).

## Dissemination

Developing, updating, and maintaining the instructional material for inquiry‐based courses is necessarily more time‐consuming than for other types of courses. For instance, laboratory manuals and bioinformatic questions must be updated each year to reflect frequent changes in electronic resources (databases change, merge with others, etc. 62). Also, to improve the curriculum's content and student experience, constructive feedback from students and TAs is taken into account at the end of each term. These modifications occur both in our in‐house laboratory manual (heavily relied on in the first semester) and in the general instructional materials that we develop (both semesters).

To disseminate BioLEd materials to both students and faculty/staff, a publically accessible resource is available at http://biochemlab.org. This website features portals for Instructors, Students, Proteins of Interest, and Collaborators (Fig. 2). The Instructors portal offers three resources: Instruction Modules, Spectrophotometric Assays, and Assessment & Evaluation Tools. (This region of the site is password‐protected; login credentials are available upon request.) The Instruction Modules section contains the eleven modules listed in Table 1, each of which provides educational materials such as lecture slides, videos, readings, sample quizzes, grading rubrics (Supporting Information 5), excerpts from our in‐house laboratory manual, and additional resources (often from the primary literature). Through the Assessment & Evaluation portal, users can access the various assessment tools we distribute to students, as well as the results of those assessments; past assessments are also available, annotated with commentary to describe changes made to the curriculum based on the assessments. These resources are intended to assist current BioLEd instructors (at UVa) as well as external faculty/staff who wish to implement some (or all) of the BioLEd laboratory curriculum at their own institution. All BioLEd materials are freely available either via the website or upon request.

## Conclusions

The biochemistry laboratory curriculum at UVa has been revamped to provide students with an authentic research experience. Because this laboratory course is required for chemistry majors specializing in biochemistry, and because over 70% of our 100+ chemistry degree recipients specialize in biochemistry each year, the revamped curriculum must be scalable to large numbers of students. (The fraction of students focusing in biochemistry has steadily climbed in recent years, and may well continue to do so.) With the newly developed BioLEd curriculum described here, a vast majority of UVa's new BS Chemistry graduates will have had a genuine research experience before graduating. Perhaps most importantly, the experience that students gain in a curriculum such as this is deeply relevant to the “real‐world” situations they will face after graduation, such as the need to work effectively in a group of individuals, towards a common goal, and without a detailed protocol or rubric. The lessons that students learn in a BioLEd‐like curriculum are general and transferable: whether they pursue graduate school, medical school, volunteer work, industry, or another calling, students can draw upon the resourcefulness and skills that they developed when learning how to search the primary literature for relevant information, effectively utilize web servers and other computational tools, logically design experiments, quantitatively analyze data and interpret results, and present their findings in a broader context and to a large audience of peers.

Importantly, we note that the research experiences gained in the BioLEd curriculum do not come at the expense of “traditional” learning: Pre‐ and post‐course tests, as well as participant self‐assessments, indicate that students are learning in our four main focus areas (Fig. 1). In addition, student grades improved in nearly all areas with each successive assignment. Finally, though developing the inquiry‐based BioLEd curriculum was a major undertaking, its modular design allows for facile implementation by other institutions that may be interested in adopting a research‐based model for undergraduate biochemistry education. To aid this, our BioLEd website freely provides course materials to all students and instructors.

## Acknowledgements

This work was funded by UVa (Dept of Chemistry, and College and Graduate School of Arts & Sciences), an RCSA Cottrell Scholar Award (LC), NSF DUE‐1044858 (LC and CM), and NSF Career awards MCB‐0845668 (LC) and MCB‐1350957 (CM). The authors thank the JCSG for providing clones for many of the POIs investigated by BioLEd students in recent years, and they thank Jennifer Doudna (UC Berkeley) for helpful discussion about a year‐long biochemistry laboratory. Many early generations of BioLEd students, TAs and other contributors are also thanked, including Sarah Elkin, Jeong Hyun Lee, Lauren Lee, Elleansar Okwei, Colin Price, and Ana Wang.

## Supporting information

Supporting Information

Supporting Information

Supporting Information

Supporting Information

Supporting Information

Supporting Information

Supporting Information

Supporting Information
