# Supplementary material for: Known structure, unknown function: An inquiry‐based undergraduate biochemistry laboratory course
Source: Biochem Mol Biol Educ. 2015 Jul 6;43(4):245–62. doi: 10.1002/bmb.20873 (PMC4758391; doi:10.1002/bmb.20873)
Supplement: Supplementary file 1 — Supporting Information [file BMB-43-245-s001.docx]

Known Structure, Unknown Function:

An Inquiry-based Undergraduate Biochemistry Lab Course

Cynthia Gray, Carol W. Price, Christopher T. Lee, Alison H. Dewald, Matthew A. Cline,

Charles E. McAnany, Linda Columbus, Cameron Mura

**Supplementary Information, 1**:

Precise learning gains, organized by course modules (see also Table I)

For each module in Table I (main text), we include here an outline of the learning gains that we expect for a student. Each assignment is graded in accord with these gains; that is, the questions or graded portions of any assignment are grouped according to learning gains (Fig 1, main text), such that we can use the assignment to help assess the learning gains for a particular topic or concept.

Module 1A: Literature searches; electronic resources and tools

This module includes a one-hour lecture that highlights the many resources and literature search engines used by biomolecular scientists. In addition, the UVa library offers a program wherein a subject librarian is invited for subject-based instruction on current assignments and projects, with a focus on article databases, plagiarism, and critically evaluating resources. The learning gains assessed are (i) overall understanding of the purpose of literature searches (aims and concepts), (ii) identification of relevant literature (broader context), and (iii) the ability to find and understand resources (aims and concepts).

Module 1B: Basics of pipetting with the micropipette

Students deliver water to a weigh-boat on a balance and determine the volume delivered (based on the measured mass and the density of water) from each of their pipets (a P1000, P200, and P20). They calculate standard deviations for each measurement. The learning gains assessed for this assignment are (i) pipetting skills (aims and concepts) and (ii) quantitative skills (data processing).

Module 2: Critically reading the primary literature

This module includes both an interactive instructional period and a follow-up assignment for the laboratory period. The goal of this module is to (i) define what the primary literature encompasses (peer review, publication frequency, citations); (ii) address the differences between the *primary* (a direct report of research results and findings), *secondary* (review articles), and *tertiary* (textbooks and references) literature sources; (iii) introduce students to active and critical reading (identify the main question being addressed in the study, the conclusions, critically evaluate the data used to support the conclusions, pinpoint any missing factors or limitations); and (iv) walk students through relevant papers (i.e., provide a guided journal-reading experience). The last two goals are achieved using the C.R.E.A.T.E. method developed by Hoskins *et al*. (ref [16] in the main text). The learning gains assessed in this module are for the student to (i) understand how the data in published articles were generated (aims and concepts), (ii) critically analyze the results of each figure in the article (aims and concepts, and data processing), (iii) elucidate a hypothesis based on the results of each data figure (experimental design), and (iv) be able to propose a follow-up experiment (experimental design).

Module 3: Biochemical buffers and solutions

This Module focuses on practical aspects of buffer preparation and introduces concepts of relevance to protein solutions (e.g., factors influencing solubility and stability, Hofmeister series, etc.). Students were taught the principles of making a buffer, using the Henderson-Hasselbalch equation, in their chemistry and biochemistry lecture courses. In this module, the *practical* considerations for buffer selection and solution preparation are emphasized—ionic strength, buffering capacity, compounds that may act as potential interferents in the reaction/assay, etc. Students are expected to (i) choose an appropriate buffer for downstream purification and enzymatic assays for their POI, (ii) calculate how to prepare the buffer, (iii) determine a reasonable volume to prepare, and then (iv) prepare the solution. The primary learning gain addressed in this module is laboratory skills. When students revisit these concepts in Module 9 (dialysis), the choice of buffer and how it is made can be assessed in terms of experimental design.

Modules 4–5: Enzyme kinetics assay (a hands-on assay using lactate dehydrogenase or a similarly well-characterized, commercially available enzyme)

This module seeks to provide students experience with (i) performing enzyme kinetics assays, (ii) the techniques used in performing spectrophotometric enzyme assays, (iii) the process of experimental design, (iv) how to process raw data, and (v) how to analyze/interpret the resultant processed data. Students are expected to perform an extensive pre-lab assignment which requires them to think deeply about the experiment before coming to lab (the learning gain here is experimental design). While a four-hour lab session suffices for performing the experiments, completing this lab requires students to arrive well-prepared. Because of their *active* role in planning and executing the lab work in this module, students develop an appreciation of the practical considerations of experimental design and preparedness, and are more likely to carefully scrutinize protocols and attempts to make improvements (‘shortcuts’), versus students who simply execute a pre-prepared protocol. In addition, students gain familiarity with each step and are thus better prepared to make logical choices when troubleshooting or adapting the protocol.

In the second kinetics module (Module 5), students learn how to analyze data from kinetics assays. Using a chosen (fixed) enzyme concentrations, students perform a series of assays in which substrate concentration is varied. They convert raw data (absorbance values) to concentrations using the appropriate extinction coefficients, generate plot(s), and calculate all possible enzyme kinetics parameters (learning gain: data processing). This module includes discussion of various means of data presentation and analysis (Michaelis-Menten, Lineweaver-Burke, Hanes-Woolf plots), determination of kinetics parameters (*K*_M_, *k*_cat_, *v*_max_), interpretation of data from inhibitor assays, and analysis of alternate substrates. We have found that special attention must be paid to (i) how to use a spreadsheet effectively (most students use Microsoft Excel, some use Origin; we do not enforce a specific program), (ii) careful calculations of concentrations, and (iii) dimensional analysis. In working-up the data, students also learn how to effectively represent quantitative data as figures, which is a skill they use extensively in the second term. When writing the lab report for these two Modules, students are expected to demonstrate their understanding of the aims of the experiment and to relate their work to a broader context. Thus, all four of our learning gains are assessed in the laboratory and assignment associated with Modules 4-5.

Module 6: Computational biology, I: Bioinformatic tools, web/database resources

This module introduces students to computational methods that are commonly used in modern biochemical research. For the lab portion of this module, we draw upon an extensive and up-to-date collection of ‘Education Articles’ published in *PLoS Computational Biology*, including a practical tutorial on using many different types of bioinformatic approaches to analyze protein function from 3D structure (see main text). This Module’s lecture materials touch upon the core ideas of (i) molecular evolution and phylogeny (including phylogenetic trees); (ii) sequence alignment methods (pairwise and multiple, substitution matrices, gaps, local/global alignment, *E*-scores); (iii) the basic idea of ‘profiles’ and functional annotation; and (iv) structural bioinformatics (pairwise structural alignment, finding evolutionarily-conserved functional ‘patches’, etc.). The last portion—3D structural analysis and an introduction to the PyMOL molecular visualization environment—supplies a natural bridge to the next computational section (Module 11).

Students perform extensive, in-depth bioinformatic analyses of their POI during lab time, and complete an assignment that details their findings. The learning gains assessed in this module include understanding aims and concepts, investigative skills, critical thinking, and broader context.

Modules 7–9: Recombinant protein expression, chromatography, protein purification, SDS-PAGE, and dialysis

This module introduces students to experimental techniques that are central to biochemical research, including two approaches deemed by the American Chemical Society (ACS) to be important general techniques: electrophoretic methodologies and chromatographic separations. This Module includes interactive lectures that cover (i) general methods for cloning recombinant proteins (so they learn how their POI plasmid was created), (ii) regulation of protein expression and induction in various plasmid vectors, (iii) the usage of chemical tags, such as (His)_6_, for purification purposes, (iv) gel-filtration, ion-exchange, and affinity chromatography, (v) electrophoretic gel separation techniques, and (vi) dialysis. We have found that supplying student groups with novel, uncharacterized proteins, which they first research via the literature and bioinformatic methods, gives students a sense of ownership of the project and instills the excitement for discovery that only true research can bestow.

Modules 7–9 span four weeks, but are contained within one lab report. This lab report allows us to assess the students in each of the four main learning gains we have identified (Fig 1). Student lab reports should (i) demonstrate an understanding of the purpose of the experiments (aims & concepts), (ii) display a grasp of the methods (experimental design), (iii) feature clear figures of carefully processed data (data processing), and (iv) relate their work to the ultimate goal of characterizing their POI (broader context).

Module 10: Protein concentration determination; ligand-binding assays

In the BioLEd curriculum, students are taught how to quantify proteins by two methods: (i) UV/vis spectroscopy (absorbance at 280nm, *A*_280_) and (ii) a modified Bradford assay that depends on Coomassie blue binding (a BioRad assay). Also, the molecular basis of protein•ligand binding are introduced in lecture slides, and methods for analyzing such data are introduced and summarized (include equilibrium dialysis, ligand-blotting, filter-binding analysis, isothermal titration calorimetry, mobility shift assays for nucleic acid-binding proteins, and spectroscopic (notably fluorescence) measurements). In past labs, students have studied the binding of the ligand Coomassie blue to bovine serum albumin (as described in ref [18] in the main text). As for the other two lab reports in the first semester, the grading rubric for this Module 10 report also addresses the four learning gains: understanding aims and concepts, experimental design, data processing, and broader context.

Module 11: Computational biology, II: Molecular visualization, modeling, docking

This Module introduces the basic concepts of molecular visualization and graphics (e.g., stereoscopic viewing, different types of molecular representations, surfaces, ‘scenes’, ray-tracing, etc.), followed by an overview of some elementary ideas of molecular modeling (e.g., rotamer libraries, homology modeling). Perhaps of greatest potential utility for their POI functional studies, we introduce the students to computational methods for ligand-protein docking; in the past we have employed the PatchDock server, and most recently we have begun introducing students directly to the Linux-based usage of the AutoDock suite (see main text). Introducing this computational Module before the second semester enables students to begin immersing themselves in the (potentially foreign) computational tools and concepts; this, in turn, leads to students (i) becoming independent practitioners of the computational methods within a matter of weeks/months, and (ii) fruitfully applying this new knowledge and computational expertise to their POI over the remainder of the year-long course. Learning gains assessed in the Module 11 assignment include data processing (to generate figures that are scientifically convincing and lucid), critical thinking (analyzing the docking results), and general biochemical knowledge (to interpret the results).
