# Supplementary material for: Known structure, unknown function: An inquiry‐based undergraduate biochemistry laboratory course
Source: Biochem Mol Biol Educ. 2015 Jul 6;43(4):245–62. doi: 10.1002/bmb.20873 (PMC4758391; doi:10.1002/bmb.20873)
Supplement: Supplementary file 2 — Supporting Information [file BMB-43-245-s002.docx]

Known Structure, Unknown Function:

An Inquiry-based Undergraduate Biochemistry Lab Course

Cynthia Gray, Carol W. Price, Christopher T. Lee, Alison H. Dewald, Matthew A. Cline,

Charles E. McAnany, Linda Columbus, Cameron Mura

**Supplementary Information, 2**:

A sample PyMOL-based in-class activity — Molecular visualization & structural analysis of serine proteases

Useful PDB IDs and other helpful resources (e.g., Proteopedia, <http://proteopedia.org>):

- 2AGI: “*The leupeptin-trypsin covalent complex at 1.14 A resolution*” (2PTN, no leupeptin)
- 2CGA: “*Chymotrypsinogen A. X-ray Crystal Structure Analysis and Refinement of a New Crystal Form at 1.8 A Resolution*”
- <http://www.proteopedia.org/wiki/index.php/Trypsin>, <http://www.proteopedia.org/wiki/index.php/Chymotrypsin>, <http://www.proteopedia.org/wiki/index.php/Elastase>


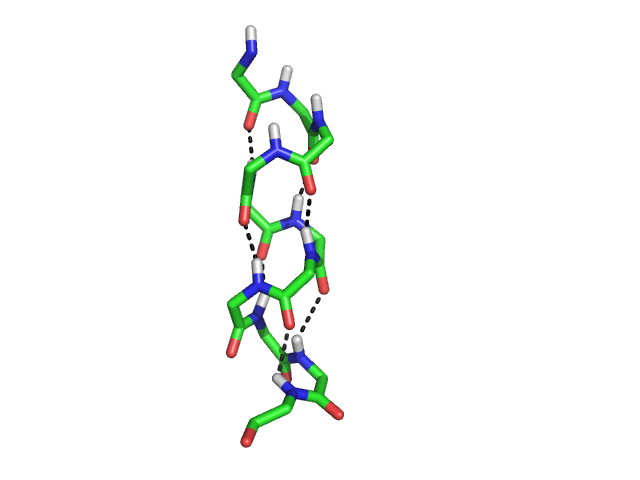


**N**

**C**

**Trypsin**

- Open the trypsin PDB file in PyMOL and then:
- Add hydrogen atoms (‘A’ pull-down menu → ‘Hydrogens’ → ‘Add’)
- Issue this command: select myhelix, resi 235:245
- Issue this command: hide everything
- Display cartoon of myhelix
- Double middle-click near the center of the helix (to center the molecule)
- Zoom the view of the helix
- Identify the N- and C-termini of the helix
- Show main chain atoms as sticks (for the helix selection)
- Set background color to white
- Save the image, and label the termini and the H-bonds that stabilize the α-helix
- Now, for the same helix, select the **hydrophobic residues** and color the selection (green). Hint: consider a command such as this (all one line):


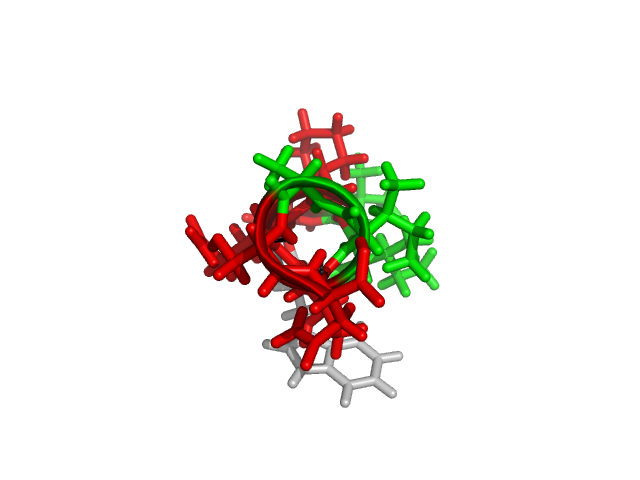

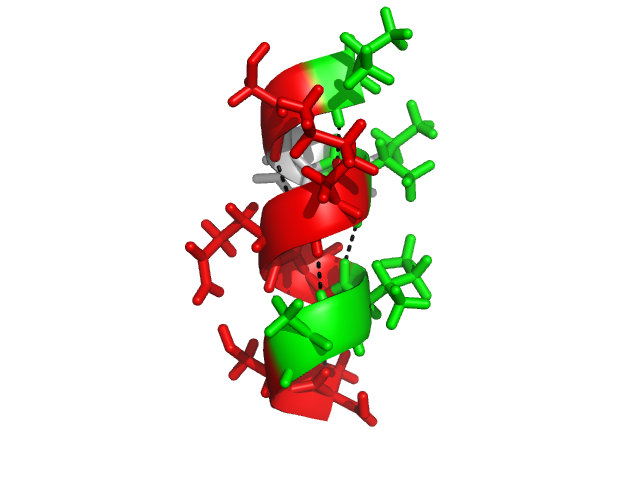


select my_hydrophobic, resn \ leu+val+ile+gly+pro+ala+phe+met

- Now select the **polar residues** and color the selection (red), using a command similar to this:

select my_polar, resn \ glu+asp+asn+gln+lys+arg+his+ser+thr

Is ‘myhelix’ amphipathic? Explain.

-
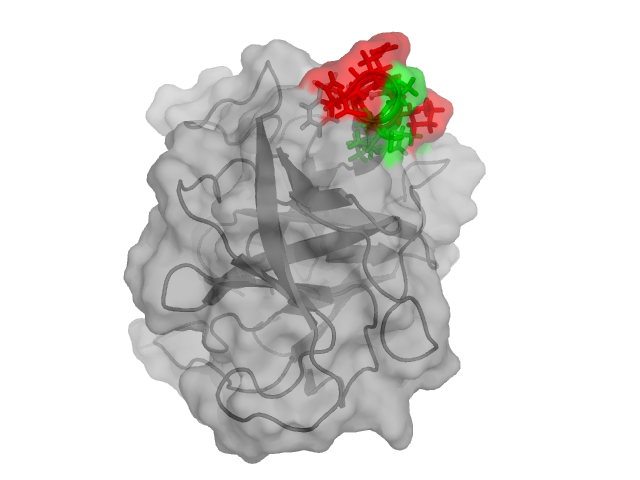
Select residues 1-234 and color them gray.
- Now display the cartoon of the entire trypsin molecule.
- Print it out and label the hydrophobic regions of the helix and explain nature of the helix with respect to the rest of the protein.

**Chymotrypsin** (chymo), another serine protease

- Open the chymo PDB file in PyMOL.
- Using the commands above, identify an amphipathic β-strand. What residues comprise the strand?


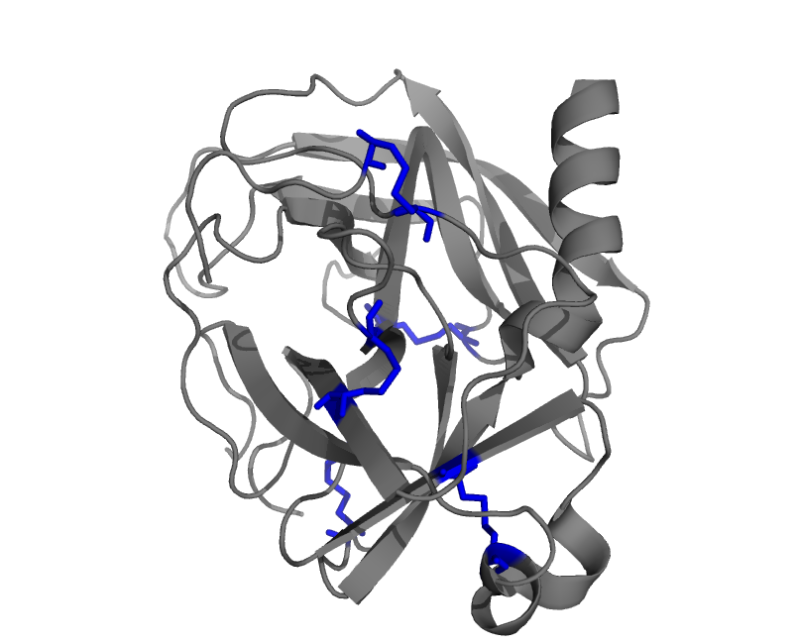


- How many disulfide bonds are there in chymotrypsin?

…The Protease Mechanism — substrate specificity!

- Select residues 57, 102, and 195 using this PyMOL command: __________________________?
- Color the entire molecule gray and the selected residues another color.
- Display a molecular surface.

*Do you see a large cavity next to the colored residues?*

- If so…
- What properties of the cavity do you believe to be important in binding the peptide substrate?
- Compare the cavity to that of trypsin and elastase. Do they differ? (If so, how?)
- Prepare a figure that illustrates the differences amongst these proteins (highlighting active site residues, cavities, and stabilizing residues).
- Select residues 215-219 and color them a different color (view with and without the surface).

What is the function of these residues in the chymotrypsin mechanism?

-
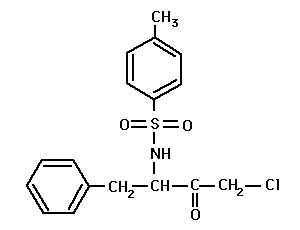
The compound tosyl-L-phenylalanine chloromethyl ketone (TPCK) specifically inhibits chymotrypsin by covalently labeling His57.
- Given the chemical structure, can you suggest a mechanism for the inactivation reaction? (You can consult the enzyme catalysis chapter in Voet & Voet or other standard *Biochemistry* texts.)
- Why is this inhibitor specific to chymotrypsin?
- Draw a derivative of TPCK that might inhibit trypsin, highlighting what moieties you’ve changed.
