# Supplementary material for: Known structure, unknown function: An inquiry‐based undergraduate biochemistry laboratory course
Source: Biochem Mol Biol Educ. 2015 Jul 6;43(4):245–62. doi: 10.1002/bmb.20873 (PMC4758391; doi:10.1002/bmb.20873)
Supplement: Supplementary file 3 — Supporting Information [file BMB-43-245-s003.pdf]

Known Structure, Unknown Function:
An Inquiry-based Undergraduate Biochemistry Lab Course

Cynthia Gray, Carol W. Price, Christopher T. Lee, Alison H. Dewald, Matthew A. Cline, Charles E. McAnany, Linda Columbus, Cameron Mura

Supplementary Information 3:
Molecular Docking Tutorial for the Biochemistry Lab (Chem4411/21)

1 Initial Setup, Introduction to Linux

In this lab, we will dock ligands to your POI using the AutoDock Vina software on the Linux operating system. This section contains some computing details that may seem superfluous at first, but the material is critical to the rest of the docking workflow, so please study it carefully.

1.1 Basic Information and Nomenclature Conventions

Here we explore the basics of using the Linux operating system, and we describe some important terminology and formatting conventions that appear throughout this tutorial. Note that new terminology is defined in context using *italics* typeface.

In the following pages, we show commands/concepts/terms in the left-hand side of the table, and matching explanations follow on the right-hand side.

Dolphin	The file manager for the Linux distribution (Fedora) and window manager (KDE) that you will be using. The Dolphin system works essentially like its Windows counterpart, Windows Explorer. To start, single-click the Home icon on the Desktop.
Konsole	A graphical environment that places you in a Unix *shell*, which allows you to input commands as text. To open a Konsole, right click on empty space on the Desktop and select `Konsole`.*
Home Directory	This is the directory that is shown when you first open Dolphin. You can consider this as roughly equivalent to My Documents in Windows. It is often denoted by a '~' in file-paths.

*The Konsole program is, technically, a *terminal emulator*, which provides you with a *command-line interface* (CLI). There are three two fundamentally distinct modes that one uses in working in Linux: (i) GUI-based (mouse clicks, like in MS Windows or Mac OS) and (ii) text-based (in the shell, using the CLI). In reality, a hybrid of (i) and (ii) is often the most efficient approach, and for this reason we introduce you to the Unix shell in this lab course. Many commands that we will use can be run only from the CLI, or can be run far more powerfully via the CLI (this may be counterintuitive, right now!). In general, running commands and performing operations in the shell will save much effort versus other methods (and is more easily reproduced, as one can communicate to someone else a list of text commands much more easily than showing mouse clicks across the graphical desktop background).

[jobDirectory]	This is how we will refer to the directory where all of your work will be done for a single *job*. (Think of a job as one small, self-contained unit of work; for example, it would be one replicate, if you were pipetting many solutions to repeat a wet-lab experiment in triplicate... In computational biology, you would say you performed the calculation, or *job*, three times.) You will create your [jobDirectory] in the next step of this tutorial, and you will need to navigate to it on several occasions.
text	We will use this formatting to highlight many words throughout the tutorial. This font indicates one of two things, depending on context. First, you are looking for a button, field or file called some_name. The other case is that you will be typing a text command using the keyboard. In both cases, it is text that you should find verbatim, unless...
[text]	Text with [square brackets around it] will be text that is not precisely the same for every use. This will be such things as PDB codes or ligand names, which will generally differ for each job.
GUI	This stands for Graphical User Interface, which is how you usually interact with your computer.
PGUI	This is a denotation that will be used when the command is in the small gray PyMOL box containing the File menu. 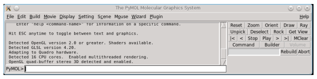
VGUI	This is a denotation that will be used when the command is in the PyMOL Viewer. Most commands given here will be on the right side panel (the graphical menu of buttons). 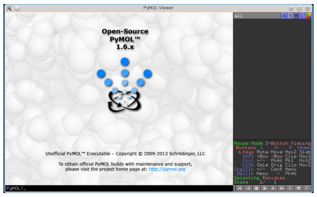
AGUI	This is a denotation that will be used when the command is to be issued in the AutoDock plugin. Make sure you check the tabs at the top, if you are having a hard time finding a button. 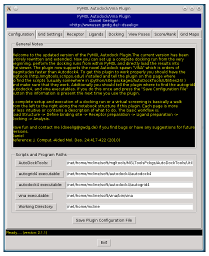

`PyMOL>[cmd]`	This indicates that you should type `[cmd]` in the PyMOL shell ( ). Feel free to use PyMOL's GUI for any commands that you feel more comfortable with, but note that it is often simpler for us to give precise instructions by using text commands, for reasons described in the footnote on page 1. (Also, as you learn PyMOL's text commands, you will become faster and more versatile in PyMOL.)
`:)[cmd]`	This indicates that you should type `[cmd]` on the Konsole command line ( ). Note that `:)` is **not** part of the actual command, but rather it denotes the **shell prompt**; so, do not type a `:)`, instead just type that text following immediately to the right of the closing parenthesis.
`:)cd [dir]`	This command, which stands for change directory, allows you to navigate the filesystem while in a shell (in *Konsole*). `[dir]` refers to the directory that you wish to go to, so to move to a directory called `foo`, you would type `:)cd foo`. Some special symbols can occur in the place of `[dir]` in order to do specific things. For example...
`:)cd ..`	A command that moves you up in the filesystem by one directory. So, if you are in `~/TM1689.mcline/test1`, then `:)cd ..` would move you to `~/TM1689.mcline`
`:)cd`	In this special case of `cd` (when no directory is specified) you will be taken back to the Home Directory.
`:)ls -l`	This extremely usful command shows a listing of all files in the current directory (analogous to seeing a list of all files graphically, in the Windows or Mac OS).
`:)pymol`	This command launches PyMOL from Konsole. Note that you can also maneuver the file-system from within PyMOL, using `cd`, in the same fashion as from within a shell (Konsole).
`<TAB>`	This denotes a literal TAB on the keyboard. We use this key often because `<TAB>` is a powerful tool when using the shell (Konsole) and from within PyMOL's command line. `<TAB>` triggers the computer to try and finish that which you began typing (this is known as *tab completion*). This means that if you have a long command name, for example `autoligand`, then, more often than not, it will suffice to begin typing `aut` and then press the `<TAB>` key and let the computer finish your thought. If there are multiple (reasonably few) options for completion of a command that begins `aut...`, then the shell will list those potential commands, and that is handy in its own right (e.g., when you know only the begining of a command, or can't remember what some file was called...).

1.2 Setting up Your Directory

In this section, you will create your `[jobDirectory]` and start PyMOL in Linux for the first time.

1. Open the home directory from the Desktop icon `Home`
2. Right click in Dolphin.
3. `Create New` → `Folder...`
4. Name the Folder `[POInumber].[userID]`, where userID is your UVa computing ID and POInumber is the ID code for your protein of interest (POI). For example, I would use `TM1698.mac7yx`
5. Enter the folder that you just created by single-clicking on it (a single click is often enough for this Linux environment; you don't necessarily need to double-click like in Windows).
6. Repeat the above method to create a new job folder called `test1` (or whatever you would like) in `[POInumber].[userID]`. This is where a single job will be performed. If a new job is done then create a new folder in `[POInumber].[userID]` to work in.

2 Performing a Docking Calculation Starting with Sanitized Receptors and MOL2 ligands

Before we can begin any docking project, we must gather the necessary files into your `[jobDirectory]`.

1. Open the Home Directory and navigate to the `RECEPTORS` directory.
2. From the receptor directory, click once on the appropriate `[receptor].pdb` file and use the Ctrl-c keystroke to copy the file (alternatively, right-click on the file to see the list of possible options, one of which should be to copy it).
3. Navigate to your `[jobDirectory]`, which is the directory we named `test1`.
4. Use Ctrl-v to paste the file into your `[jobDirectory]`.
5. Now navigate to the `LIGANDS` directory in the Home Directory and repeat the copy-and-paste procedure for the `[ligand].mol2` file.
6. Open Konsole and navigate to your `[jobDirectory]`, like so: `:)cd [jobDirectory]`.
7. Open PyMOL from within this Konsole by typing `:)pymol`.

Now, we can set-up and run our docking calculation from within PyMOL using the following sequence of operations in the PGUI window:

`PyMOL> load [ligand].mol2`	For the ligand, we are using the MOL2 coordinate file downloaded from the ZINC database. If you ever need coordinates for a ligand, we advise that you start searching at the ZINC database (an online database of purchasable ligands), as your search may well end there or never be complete. Note that no pre-processing has been performed on the ligand prior to your receiving it here.

`PyMOL> load [receptor].pdb`	We use Protein Data Bank (PDB, `.pdb`) structure files for docking. The PQR format (that was also in `RECEPTORS`) is useful in electrostatics calculations, but that can be a topic for later analysis or discussion.
`PyMOL> h_add [receptor]`	This command adds hydrogens based on empty valences. (As a sidenote, the PyMOL protonation tool is necessarily the 'best' algorithm, but its advantages are that it does not require other third-party programs or libraries, and it isn't as picky about ligands in the PDB file (versus other methods).
PGUI: `Plugin` → `Autodock/Vina`	This opens the GUI plugin that we will use to set-up our docking calculations. The GUI should open to the `Configuration` tab, which should already be set with appropriate parameters.
AGUI: `Grid Setting`	In this tab, we will set-up the three-dimensional (3D) region of space where the molecular docking will occur.
AGUI: In `Calculate Grid Center by Selection`, type `[receptor]` in the field. Press Enter.	This places the center of the calculation grid (last step) at the center-of-mass of your protein, which is a good starting point; we may end-up needing to adjust this in a moment (see below).
AGUI: In `Parameters`, change `Spacing` to `1.000`.	In AutoDock Vina, a 3D grid is laid over the protein, and the interaction energy of various atoms is computed at each grid point. By setting the spacing to 1.0, the other measures presented in the GUI will also be in units of Angstroms (Å), and thus more easily understood. (If this step is confusing, that is ok: the PyMOL plugin is smart enough to adjust your measurements to correct geometric amounts when it creates the configuration file.)
AGUI: In `Parameters`, adjust `X-points`, `Y-points` and `Z-points` until the grid box covers the protein.	In this step, you are telling AutoDock Vina where to search for the potential ligand-/substrate-binding site. For faster calculations, you will want this grid to be as small as possible. You can also adjust the `Grid Center Coordinates` to help shrink this region.
AGUI: In `Config File`, press `Save`	This saves the coordinates for the grid to a file called `config.txt`. IMPORTANT: This is probably the first time so far that it has become crucial to have done all of the work in **your** `[jobDirectory]`. If, instead, you had been working in the Home Directory (where multiple users where working), then only one `config.txt` would have been saved in the Home Directory (the others would be over-written), and so odds are it isn't yours! This can generate great confusion.

AGUI: `Receptor`	In this tab, we will finish preparing the receptor for docking by saving AutoDock's own special format, PDBQT, which stores some additional information (beyond the coordinates in the PDB file format). The most critical piece of additional data is the bonding information for all atoms in the system — this information defines the molecular *topology* and also enables us to specify which bonds we will allow to freely rotate.
AGUI: Select `[receptor]` from the PyMOL selections.	Here, you are simply telling PyMOL which of the objects that it is storing is the *receptor* (i.e., your POI, which is to be docked to).
AGUI: Press `Generate Receptor` ->	The PyMOL plugin will now go find the correct preparation script and will apply it to the receptor (your POI). So, just wait for it to finish and add your receptor to the `Receptors` list. While this is occurring, you should look in the `Log` field for any errors, because if any part of this setup was wrong then this step is likely to fail (not to worry, this is probably not your fault). Unfortunately, these error messages can be subtle and, sometimes, the program will continue on computing, but will give flawed results. If an error arises here, and if you research it a little (use Google) and do not understand it, please show your TA the error message (it may be a computer/IT problem that can be readily addressed by one of us).
AGUI: `Ligands`	This is exactly the same as the receptor (above), except that now you are chosing your *ligand*... So, give this a shot on your own.
AGUI: `Docking`	This is where we can print the final configuration file for AutoDock Vina. The `Run Vina` button seems to be broken (software is not always perfect), and so we will have to resort to the Konsole to actually run Vina.
AGUI: Press `Write Vina Input File(s)`	The program writes another file in your `[jobDirectory]`, which is probably starting to look like a cluttered mess. That's OK.
Open a new Konsole and `:)cd [jobDirectory]`.	There is a shortcut to do this, actually: In the Konsole that is running PyMOL, double-click the free area at the bottom, located beside the current tab. This opens a new tab which provides a shell that is already in the directory of the previous tab (so you don't have to navigate there again).

`:)vina --config [ligand].vina_config.txt`	By executing this command — type it exactly as shown, and press Enter — your computer should happily begin computing docking conformations. When this finishes, we will begin the fun part, *analysis* of the docked structures of the ligands to your POI (each of these are known as docking *poses*). Wait for this job to run to completion, which will be apparent when the command prompt `:)` returns control to you (the user) rather than the program that just finished running.
`:)dockProc -csv log.csv [ligand].vina.log`	This executes an in-house *script* that reads the log file and builds a simple table in comma-separated value (CSV) format; MS Excel or most other data-processing/math software can read/import such files.
Move `receptor.[receptor].pdb`, `[ligand].docked.pdbqt` and `log.csv` to your computer (e.g., you can email it to yourself).	Analysis of the docking results does not require the plugin nor any Vina software, so we can complete that stage of our work on any computer workstation with PyMOL installed (e.g., your laptops). Make sure that you save these results in a place that you can find. If you prefer to continue on the Linux platform, we have several workstations in the research lab that you can ask us about using.

3 Analyzing Docking Results: The Mechanics

In this section, we will load the docked ligand conformations (the poses) into PyMOL for further analysis... and that will be all that is covered in this current tutorial, because analysis of the docked poses — literally, the docking results — is your job, and is specific to your POI. (Note that by ‘analysis’ we mean visual analysis and interpretation of the locations of the ligands [on the POI], their detailed 3D structures, inter-atomic interactions, ligands$\cdots$POI contacts, etc.)

1. Open `log.csv` in Excel or a comparable program. If you are using a Linux workstation, we suggest LibreOffice Calc. Note that there are no headings. This is because we wish for this file to be easily loaded into any program that accepts CSV, but headings may hinder such compatibility. The headings are, from right to left, ‘Ligand Identifier’, ‘Mode Number’, ‘Binding Affinity (kcal/mol)’, ‘RMSD upper bound (Å)’ and ‘RMSD lower bound (Å)’. The RMSD values are of limited value, particularly when you dock to the entire protein (known as *blind docking*). The binding affinity can be viewed (very roughly) as the thermodynamic binding affinity, were the ligand to bind in exactly that pose; however, these are not truly accurate ΔG°_{bind} values, and are only particularly useful when internally compared across different docked conformations/ligands/etc.

2. Start PyMOL on your computer.

3. Use the PGUI: `File` → `Open...` to find and load in your `receptor.[receptor].pdb` and your `[ligand].docked.pdbqt`

4. The docked poses are now in one PyMOL object with multiple states. To switch between the states you can use the arrows at the right of the PyMOL Viewer.

Congratulations! Now that you're familiar with the mechanics of a docking calculation, use what you already know about PyMOL and biochemistry to draw conclusions from the docked conformations; ideally, perhaps you will be able to assess the ligand-binding preferences of your POI.

Appendices

PyMOL: A Quick-start Guide

Installation

There are two major ways to install PyMOL. First, one can obtain the educational version, though that edition is some releases behind the latest production version. Nevertheless, the educational version can be installed via a relatively simple process, and if you wish to use this version, Google 'pymol' (follow the directions at http://www.pymol.org). The second method requires you to compile PyMOL from source-code; this considerably more complicated route does provide you with the very latest, 'bleeding-edge' version. For Windows, visit http://www.pymolwiki.org/index.php/Windows_Install for directions. For Apple, go to http://www.pymolwiki.org/index.php/MAC_Install. Both methods may take some tinkering and online searching in order to make sure that appropriate libraries are in-place, cross-compatibility with versions of the Python and Tcl programming languages is not a problem, and so on.

Navigating in PyMOL

Object	This organizational unit is how PyMOL internally stores a 3D structural entity. When a protein or any other molecule is opened in PyMOL, that auto-creates one *object*; the next molecule that is loaded will be a new object, and so on. These objects can be edited as one group.
Object Control Panel	This is the area on the right-hand side of the PyMOL Viewer providing a list of the objects. Many of the GUI commands will be found here, and we will assume that you can explore this area on your own.
Left-click & drag	This rotates the protein representation in 3D space. Play with this for awhile to become comfortable with how this works.
Right-click & up-down drag	This zooms in and out on the protein.
Scroll wheel	This changes the *clip*, which is the width of a slab that dictates how much depth of the 3D space (the z-direction) is rendered at once. Most of the time, it's not a bad idea to begin by increasing the clip until the entire protein can be seen (see also PyMOL's closely related 'zoom' and 'center' commands). Another way to acheive this is to type `zoom` in the PyMOL PGUI.

`PyMOL>load [file]`	This loads a structure file (e.g., in PDB format) into PyMOL, and thereby *instantiates* a new object corresponding to this structure.
`PyMOL>save [file], [selection]`	This is the command for all of PyMOL save functionality, so it is a bit intricate. First, you specify the `[file]`, which is what the file will be called. This needs the extension because that is how PyMOL determines in what file format to save. The two important types are `.pse`, which is a PyMOL session file allowing you to save your work, and `.pdb`, which simply specifies a 3D structure in PDB format.
`fetch [pdbCode]`	This automatically retrieves the PDB entry from the PDB database, without your having to explicitly download it first (in fact, on Linux the PDB file will be downloaded to the local directory from which PyMOL was launched).
`PyMOL>orient [object]`	This resets the view to see the `[object]`.
`PyMOL>delete [object]`	This removes the object from PyMOL.

Selections in PyMOL

Atom selections are a vital part of being able to manipulate molecules and subsets of molecules in PyMOL (or any other molecular visualization software environment). For high-quality molecular graphics, you will have to become quite familiar with *named atom selections*. Selections can be thought of as a type of object, but can contain any logical set of atoms, which can then be manipulated together as a unit (by 'logical' we mean in a Boolean sense). You can make selections with text commands or by clicking on the protein. The click method has seven modes for different selection *scopes*: atoms, residues, chains, segments, objects, molecules and C-alphas. To change the mode, PGUI: `Mouse` → `Selection Mode`.

`PyMOL>select [selectionName], [descriptors]`	This is a command that makes a selection in PyMOL using logical descriptions. The `[selectionName]` is what the selection will be called in the Object Control Panel, and `[descriptors]` is the logic statement for whether or not an atom belongs in the selection. How to form the logic statements will be the rest of the topic of this section. If the `[selectionName]` is omitted, then the name will default to simply 'sele'.
`[object]`	When an object is included as part of the descriptor, then an atom must be part of that object in order to be chosen. So, if you would like to select all atoms in your receptor, the simple command would be `PyMOL>select sele, [receptor]`. This isn't useful in and of itself, but will often be used in logic statements (when multiple objects are loaded, e.g., your POI and a homolog to be used for structural alignment in PyMOL).

resn	This is a descriptor that means residue name. So, if you wish to select all atoms associated with a residue that is named 'PLP' (in the PDB file from which the object arose), then the command would be `PyMOL>select sele, resn PLP`.
index	This is a descriptor that means atom index number. So, if you wish to select atom 1 of the protein then the command is `PyMOL>select sele, index 1`. For early work, this descriptor is likely not as useful as others, because the mouse can achieve the same functionality (without your having to know the atomic index number(s)).
resi	This is a descriptor that means residue identifier. So, if you would like to select residue 1 of the protein, then `PyMOL>select sele, resi 1`. Again, this may not be as useful initially because the mouse can accomplish much the same without your needing to know the residue identifiers. However, a useful feature here is the ability to use this descriptor to select either a contiguous range of residues (e.g., 'resi 1-10') or a disconnected set of residues (e.g., 'resi 1,3,5,7')
symbol	This is a descriptor that means chemical symbol. So, if you seek to select all nitrogen atoms in an object, then `PyMOL>select sele, symbol N`.
chain	This is a descriptor that choses all atoms at the chain level. So, if you would like to select all (all atomic entities) in chain A, then issue the command `PyMOL>select sele, chain A`. This descriptor becomes a usedful part of the selection logic when dealing with oligomeric (multi-chain) objects, such as is the case with many POIs.
hetatm	This special descriptor symbolizes every atomic entity in the object that is not part of the protein – i.e., is not proteinaceous (e.g., water molecules, bound ions, etc.). The simplest example of a command using this descriptor is `PyMOL>select sele, hetatm`. This selection *macro* gets its name from the fact that 'hetatm' is the starting string in these non-amino acid lines in PDB files.
not	This operator modifies an otherwise 'normal' atom selection string by (logically) negating it. An example would be `PyMOL>select sele, not hetatm`, which would select all non–hetero-atoms (i.e., the protein).
and	This boolean logical operator combines two descriptors by selecting only those atomic entities that satisfy both descriptors (i.e., it is the logical *intersection*). An example would be `PyMOL>select sele, [object] and symbol C`, which would select all of the carbon atoms in `[object]`.

or	This boolean logical operator combines two descriptors by selecting only those atomic entities that satisfy at least one of the descriptors (i.e., it is the logical *union*). An example would be `PyMOL>select sele, symbol N or symbol O`, which would create an atom selection containing all of the oxygen and nitrogen atoms in the object.

Modifying the Molecular Scene/Representation

`PyMOL>color [color], [selection]`	This colors the selection to the `[color]`. The GUI can be used to determine which colors are available, and then this command can be used to then chose a particular color (by name).
`PyMOL>util.cbag [object]`	This colors the atoms of the `[object]` with carbon = green, oxygen = red, and nitrogen = blue.
`PyMOL>show [representation], [selection]`	This shows the representation of the selection. Note that it just adds the representation to shown representations, it does not remove representations. Use the GUI to find the different available representations then use this as a quick method to get back to that representation.
`PyMOL>hide [representation], [selection]`	This hides the representation of the molecule. Note that it just removes the one representation. A common command that one might use is `hide everything, [selection]`. This removes all the representations from the active display, giving you a clean slate to work with.
`PyMOL>bg_color [representation]`	This sets the background color, and is mostly used to set the background to white for making images for presentations and papers. Many people find a black background more visually appealing and simpler to work with for ‘zoomed-in’, detailed analysis of a molecular scene (better contrast); a white background is often used at a more global level (at the level of protein chains in an oligomer) and is almost always used for final rendering for purposes of a manuscript, poster, presentation, etc. (less ink used in printing a poster with molecular graphics on white backgrounds).
`PyMOL>set [name], [value], [selection]`	This is a subtle and highly flexible command that is can be used to vary literally any of PyMOL’s hundreds (to thousands) of parameter settings. Some useful particularly useful settings to consider modifying/customizing are noted below.
`PyMOL>set transparency, [value], [selection]`	This adjusts the transparency of any surfaces that are rendered (whether they are actively showing or hidden). The value ranges between 0 (full opacity; the default) and 1 (full transparency).
`PyMOL>set surface_color, [color], [selection]`	This changes the color of the surface for the named atom selection.

`PyMOL>set sphere_transparency, [value], [selection]`	Same as transparency (above), but adjusts the opacity of any sphere representations, instead of surfaces.
`PyMOL>ray [width]`	This initiates *ray-tracing* of the molecular scene that is actively visible in the viewer window, yielding high-quality, photorealistic images. `[width]` specifies the width (in pixels) of the final ray-traced output image (which is written to disk via the 'png' command).

For more information of molecular visualization and graphics, you can see "An Introduction to Biomolecular Graphics" by Mura *et al.* [1] Note that if any of the results obtained via the procedure described here are included in later work, then the convention is that you will need to cite the software used — e.g., AutoDock Vina, the AutoDock PyMOL plugin, AutoDockTools-4 (which operates behind the scenes in much of what was described above), and PyMOL. The appropriate references are [2, 3, 4, 5].

References

[1] C. Mura, C. M. McCrimmon, J. Vertrees, and M. R. Sawaya. An introduction to biomolecular graphics. *PLoS Comput. Biol.*, 6(8), 2010.

[2] Oleg Trott and Arthur J. Olson. AutoDock Vina: Improving the speed and accuracy of docking with a new scoring function, efficient optimization, and multithreading. *Journal of Computational Chemistry*, 31(2):455–461, 2010.

[3] Daniel Seeliger and Bert L. Groot. Ligand docking and binding site analysis with PyMOL and Autodock/Vina. *Journal of Computer-Aided Molecular Design*, 24(5):417–422, 2010.

[4] Garret M. Morris, Ruth Huey, William Lindstrom, Michel F. Sanner, Richard K. Belew, David S. Goodsell, and Arthur J. Olson. AutoDock4 and AutoDockTools4: Automated docking with selective receptor flexibility. *Journal of Computational Chemistry*, 30(16):2785–2791, 2009.

[5] Schrödinger, LLC. The PyMOL molecular graphics system, version 1.3r1. August 2010.
