# Supplementary material for: Known structure, unknown function: An inquiry‐based undergraduate biochemistry laboratory course
Source: Biochem Mol Biol Educ. 2015 Jul 6;43(4):245–62. doi: 10.1002/bmb.20873 (PMC4758391; doi:10.1002/bmb.20873)
Supplement: Supplementary file 4 — Supporting Information [file BMB-43-245-s004.docx]

| Your name: |  | | | |  | | | | | | | |
| --- | --- | --- | --- | --- | --- | --- | --- | --- | --- | --- | --- | --- |
| For items 1–3, rate each group member (including yourself) on the group evaluation criteria listed below. Use the following scale: | | | | | | | | | | | | |
| 1 = poor | | | 2 = marginal | | | 3 = satisfactory/average | | | 4 = good | | 5 = excellent | |
| Group evaluation criteria | | self-evaluation | | member 1 name: | | | member 2 name: | member 3 name: | | member 4 name: | | member 5 name: |
| 1. Participated in group meetings | |  | |  | | |  |  | |  | |  |
| 1. Cooperated with group; supported group process | |  | |  | | |  |  | |  | |  |
| 1. Demonstrated consistent commitment and effort | |  | |  | | |  |  | |  | |  |
|  | |  | |  | | |  |  | |  | |  |
| List a skill that each student brings to the group (e.g., bioinformatics, writing, bench-work, interpersonal skills, etc.).   \| Gray *et al*. (2014) \| Supp Info 4, p 1 of 1 \| \| --- \| --- \| | |  | |  | | |  | \| Supplementary Information 4: Sample Effort Report \| *Known Structure, Unknown Function: A New Biochemistry Lab* \| \| --- \| --- \| | |  | |  |
| List a skill which is lacking from your group overall, or which could be improved. | |  | | | | | | | | | | |
| List something specific that the group learned from you, that they may not have learned otherwise. | |  | | | | | | | | | | |
| Overall, how effectively did your group work together on this task/assignment? | |  | | | | | | | | | | |
| Suggest one change the group could make to improve its performance. | |  | | | | | | | | | | |
