# Supplementary material for: Known structure, unknown function: An inquiry‐based undergraduate biochemistry laboratory course
Source: Biochem Mol Biol Educ. 2015 Jul 6;43(4):245–62. doi: 10.1002/bmb.20873 (PMC4758391; doi:10.1002/bmb.20873)
Supplement: Supplementary file 5 — Supporting Information [file BMB-43-245-s005.docx]

Known Structure, Unknown Function:

An Inquiry-based Undergraduate Biochemistry Lab Course

Cynthia Gray, Carol W. Price, Christopher T. Lee, Alison H. Dewald, Matthew A. Cline,

Charles E. McAnany, Linda Columbus, Cameron Mura

**Supplementary Information, 5**:

Sample grading rubric from the first term

Labs 7-9: Recombinant Protein Expression, Chromatography and SDS-PAGE, and Dialysis

**Abstract**

Identify Problem Studied 2.5 pt: __________

* Isolation of protein

Mention Techniques Used 2.5 pt: __________

* Chromatography, SDS gels

Relevant Data w/ significance 2.5 pt: __________

* Information learned about protein

Conciseness 2.5 pt: __________

(Total: 10 points)

**Introduction**

Student understands aims and concepts of the experiment

Overall Clarity 4 pt: __________

Cloning/Expression of Recombinant Protein (8 pts)

Vector and Antibiotic Selection 2 pt: __________

Transformation 2 pt: __________

*E. coli* as an expression host 2 pt: __________

Induction with arabinose 2 pt: __________

Chromatography (16 pts)

General explanation of chromatography 4 pt: __________

Gel filtration

*Separation based on size 2 pt: ___________

*Explanation of method 2 pt: ___________

Ion exchange

*Separation based on pI 2 pt: ___________

*Explanation of method 2 pt: ___________

Affinity

*Separation based on a specific interaction 2 pt: ___________

*Explanation of method 2 pt: ___________

SDS-PAGE (6 pts)

What does SDS-PAGE do? 3 pt: __________

How does SDS-PAGE work? 3 pt: __________

Protein of Interest (6 pts)

Presence and specifics of any affinity tags 2 pt: __________

Theoretical MW 1 pt: __________

Theoretical pI 1 pt: __________

Identity 2 pt: __________

(Total: 40 points)

**M&M: Student understands experimental design**

Cloning/Expression of Recombinant Protein (3 pts)

Plasmid and cell line 1 pt: __________

Media and antibiotic 1 pt: __________

Inducer (IPTG, arabinose, etc.) 1 pt: __________

Chromatography (3 pts)

Gel Filtration 1 pt: __________

*Sephadex G-100 resin, lysis buffer for elution

Ion Exchange 1 pt: __________

*DEAE (anion exchange) resin, step elution with increasing [NaCl]

Affinity 1 pt: __________

*Ni-NTA resin, elute with imidazole

SDS-PAGE (2 pts)

*BioRad Ready SDS-PAGE (10-20% Tris-HCl) gel 2 pt: __________

Dialysis (2 pt) (Should be in the chromatography section, but optionally can be its own section.)

One sentence stating that protein was dialyzed into a new buffer 2pt: __________

(Total: 10 points)

**Results: Student understands data processing**

Overall Clarity 2 pt: __________

Cloning/Expression of Recombinant POI (7 pts)

SDS-PAGE gel image

*Lanes labeled 1 pt: ___________

*MW marker labeled 1 pt: ___________

*Arrow/circle to indicate induction band (**on each gel!**) 1 pt: ___________

Figure caption/legend 2 pt: __________

Text describing results and reference to figure 2 pt: __________

Chromatography (21 pts)

*Gel Filtration - 7 pts*

SDS-PAGE gel image

*Lanes labeled (incl. where blue dextran and cyt c were observed) 1 pt: ___________

*MW marker labeled 1 pt: ___________

Figure caption/legend 2 pt: __________

Text describing results and reference to figure 3 pt: __________

*Ion Exchange - 7 pts*

SDS-PAGE gel image

*Lanes labeled 1 pt: ___________

*MW marker labeled 1 pt: ___________

Figure caption/legend 2 pt: __________

Text describing results and reference to figure 3 pt: __________

*Affinity - 7 pts*

SDS-PAGE gel image

*Lanes labeled 1 pt: ___________

*MW marker labeled 1 pt: ___________

Figure caption/legend 2 pt: __________

Text describing results and reference to figure 3 pt: __________

(Total: 30 points)

**Discussion: Student capable of interpreting data and placing in a broader context**

Cloning/Expression results 3 pt: __________

*Was transformation and induction successful? How do they know?

Gel Filtration 8 pt: __________

*Did protein elute in expected fraction? Why or why not?

*Oligomer in void volume

Ion Exchange 8 pt: __________

*Did protein elute in expected fraction? Why or why not?

*Dicussion of protein pI

Affinity 8 pt: __________

*Did protein elute in expected fraction? Why or why not?

*Discussion of the His-Tag

Comparison of the three chromatography methods (yield and purity) 10 pt: ___________

Additional citations and outside research 5 pt: ___________

*How to improve purification, what can be done after purification

Error Analysis (That is, they attempt to explain why they did not get the results that they may have expected based on what they know about their protein (oligomeric state, pI, etc.) They don’t just say “It didn’t work as expected.” 3 pt: __________

(Total: 45 points)

**Conclusion**: Summarize Results 4 pt: __________

Place results in a broader context 4 pt: __________

No introduction of new data/information 2 pt: __________

(Total: 10 points)

**References** 5 pt: __________

Grand Total of 150 pt: __________
