# Supplementary material for: Known structure, unknown function: An inquiry‐based undergraduate biochemistry laboratory course
Source: Biochem Mol Biol Educ. 2015 Jul 6;43(4):245–62. doi: 10.1002/bmb.20873 (PMC4758391; doi:10.1002/bmb.20873)
Supplement: Supplementary file 6 — Supporting Information [file BMB-43-245-s006.docx]

Known Structure, Unknown Function:

An Inquiry-based Undergraduate Biochemistry Lab Course

Cynthia Gray, Carol W. Price, Christopher T. Lee, Alison H. Dewald, Matthew A. Cline,

Charles E. McAnany, Linda Columbus, Cameron Mura

**Supplementary Information, 6**:

Sample student assessment of their learning gains (SALG) survey questions

SALG Survey Questions

Instructions to students:

- Teachers value student feedback, which is taken into account when improving courses such as this one. Please be as precise as you can in your answers. Please choose "not applicable" for any activity you did not do. You may find one or more questions at the end of each section that invite an answer in your own words. Please comment candidly, bearing in mind that future students will benefit from your thoughtfulness. Remember that this is an anonymous survey: your teacher will never know what any individual student has written.
- You may see the following note next to some questions:

"D" — Department question. The department head can view the responses to these questions.

## Understanding

| 1. Presently, I understand... |  | | | | | |
| --- | --- | --- | --- | --- | --- | --- |
| 1.1 The following concepts that will be explored in this class | **not applicable** | **not at all** | **just a little** | **somewhat** | **a lot** | **a great deal** |
| 1.1.1 Literature searches and electronic resources |  |  |  |  |  |  |
| 1.1.2 Reading primary literature |  |  |  |  |  |  |
| 1.1.3 Critiquing primary literature |  |  |  |  |  |  |
| 1.1.4 Writing primary literature |  |  |  |  |  |  |
| 1.1.5 Bioinformatics tools and methods |  |  |  |  |  |  |
| 1.1.6 Molecular visualization |  |  |  |  |  |  |
| 1.1.7 Molecular modeling |  |  |  |  |  |  |
| 1.1.8 Molecular docking |  |  |  |  |  |  |
| 1.1.9 Buffer solutions |  |  |  |  |  |  |
| 1.1.10 Kinetic assays |  |  |  |  |  |  |
| 1.1.11 Enzyme kinetics |  |  |  |  |  |  |
| 1.1.12 Data analysis |  |  |  |  |  |  |
| 1.1.13 Recombinant protein expression |  |  |  |  |  |  |
| 1.1.14 Chromatography |  |  |  |  |  |  |
| 1.1.15 Protein purification |  |  |  |  |  |  |
| 1.1.16 SDS-PAGE |  |  |  |  |  |  |
| 1.1.17 Dialysis |  |  |  |  |  |  |
| 1.1.18 Protein concentration determination |  |  |  |  |  |  |
| 1.1.19 Protein-ligand binding |  |  |  |  |  |  |
| 1.1.20 Experimental design |  |  |  |  |  |  |
| 1.1.21 Choosing appropriate controls |  |  |  |  |  |  |
| 1.1.22 Systematic perturbation of an experiment to test my hypothesis generated by initial data |  |  |  |  |  |  |
| 1.1.23 Poster preparation |  |  |  |  |  |  |
| 1.1.24 Poster presentation |  |  |  |  |  |  |
| 1.2 The relationships between the concepts listed above |  |  |  |  |  |  |
| 1.3 How ideas we will explore in this class relate to ideas I have encountered in other classes within this subject area |  |  |  |  |  |  |
| 1.4 How ideas we will explore in this class relate to ideas I have encountered in classes outside of this subject area |  |  |  |  |  |  |
| 1.5 How studying this subject helps people address real world issues |  |  |  |  |  |  |
| 1.6 What do you expect to understand at the end of the class that you do not know now? |  | | | | | |

## Skills

| 2. Presently, I can... | **not applicable** | **not at all** | **just a little** | **somewhat** | **a lot** | **a great deal** |
| --- | --- | --- | --- | --- | --- | --- |
| 2.1 Find articles relevant to a particular problem in professional journals or elsewhere |  |  |  |  |  |  |
| 2.2 Identify patterns in data |  |  |  |  |  |  |
| 2.3 Recognize a sound argument and appropriate use of evidence |  |  |  |  |  |  |
| 2.4 Write documents in discipline-appropriate style and format |  |  |  |  |  |  |
| 2.5 Work effectively with others |  |  |  |  |  |  |
| 2.6 Prepare and give oral presentations |  |  |  |  |  |  |
| 2.7 What do you expect to be able to do at the end of the course that you cannot do now? |  | | | | | |
| 2.8 Please comment on how you expect this material to integrate with your career and/or life. |  | | | | | |

## Attitudes

| 3. Presently, I am... | **not applicable** | **not at all** | **just a little** | **somewhat** | **a lot** | **a great deal** |
| --- | --- | --- | --- | --- | --- | --- |
| 3.1 Enthusiastic about the subject |  |  |  |  |  |  |
| 3.2 Interested in taking or planning to take additional classes in this subject |  |  |  |  |  |  |
| 3.3 Confident that I understand the subject |  |  |  |  |  |  |
| 3.4 Willing to seek help from others (teacher, peers, TA) when working on academic problems |  |  |  |  |  |  |
| 3.5 Please comment on your present level of interest in this subject. |  | | | | | |
| 3.6 Why did you choose to take this class? |  | | | | | |

## Integration of Learning

| 4. Presently, I am in the habit of... | **not applicable** | **not at all** | **just a little** | **somewhat** | **a lot** | **a great deal** |
| --- | --- | --- | --- | --- | --- | --- |
| 4.1 Connecting key ideas I learn in class with real research scenarios. |  |  |  |  |  |  |
