# Supplementary material for: Known structure, unknown function: An inquiry‐based undergraduate biochemistry laboratory course
Source: Biochem Mol Biol Educ. 2015 Jul 6;43(4):245–62. doi: 10.1002/bmb.20873 (PMC4758391; doi:10.1002/bmb.20873)
Supplement: Supplementary file 7 — Supporting Information [file BMB-43-245-s007.docx]

Known Structure, Unknown Function:

An Inquiry-based Undergraduate Biochemistry Lab Course

Cynthia Gray, Carol W. Price, Christopher T. Lee, Alison H. Dewald, Matthew A. Cline,

Charles E. McAnany, Linda Columbus, Cameron Mura

**Supplementary Information, 7**:

Sample post–course survey questions

Hello: You are invited to participate in this post-course survey to help assess and evaluate Chem4411/21, Biochemistry Labs I & II. The questionnaire should take approximately 20 minutes to complete. Your participation in this study is completely voluntary. There are no foreseeable risks associated with this project. However, if you feel uncomfortable answering any particular questions (unless marked as required), you may skip the question. You can withdraw from the survey at any point. It is very important for us to learn your opinions. Your survey responses will be strictly confidential, and data from this research will be reported only in aggregate. Your information will be coded and will remain confidential. If you have questions at any time about the survey or the procedures, you may contact Cindy Gray by email ([cg4eq@virginia.edu](mailto:cg4eq@virginia.edu)). Participation in this survey will enter you into the lottery system described in the preliminary announcement email (we will contact you if you have won a prize from our lottery!). Thank you very much for your time and support. Please start with the survey now by clicking the ‘Continue’ button below.

Current Occupation

|  |
| --- |

For Chem4421 (second semester), who was your Teaching Assistant (TA)?

1. Abelin, Sarah
2. Dawidowski, Alison
3. Ebmeier, Jennifer
4. Fox, Donald
5. Kabzinski, Joseph
6. Kroncke, Ryan
7. Lo, Brett
8. Malaker, Tracy
9. Oliver, Ronald
10. Patterson, Peter
11. Randolph, Jennifer
12. I don't remember

Prior to these courses, did you have any experience in a research-based laboratory?

1. no
2. yes, but only as a course
3. yes, but only as an occupation/internship
4. yes, both as a course and an occupation/internship

What was your overall grade in the courses?

1. A
2. B+
3. B
4. B-
5. C+
6. C
7. C-
8. D+
9. D
10. D-
11. F
12. Prefer Not to Answer

What are your plans, if any, for science education beyond your undergraduate degree?

1. Ph.D. in biology–related field
2. Ph.D. in chemistry–related field
3. Ph.D. in physical science*
4. M.A. in life science*
5. M.A. in physical science*
6. Advanced degree in field other than sciences
7. Medical School (MD)
8. MD/PhD*
9. Other health profession
10. Law or business degree
11. Teaching
12. Peace Corps or similar
13. Work first
14. No school after college, science career
15. No school after college, non-science related career
16. Other

How did the research experience in these courses influence your postgraduate plans?

1. I had a plan for postgraduate education that has not changed.
2. It helped confirm of my postgraduate education consideration.
3. It changed my prior plan in the direction toward a postgraduate education.
4. It changed my prior plan in the direction away from a postgraduate education.
5. I still do not have plans for postgraduate education.

The following statements refer to the poster presentation portion of the courses. Please rate how much you agree/disagree with the following statements:

|  | Strongly Disagree | Disagree | Agree | Strongly Agree | Not Sure |
| --- | --- | --- | --- | --- | --- |
| Composing the poster helped me prioritize the data of my research. | ❏ | ❏ | ❏ | ❏ | ❏ |
| Presenting the poster developed my oral scientific communication. | ❏ | ❏ | ❏ | ❏ | ❏ |
| The poster presentations made me more confident in my research. | ❏ | ❏ | ❏ | ❏ | ❏ |
| Overall, the introduction of poster presentation to the courses gave me a deeper understanding of biochemistry. | ❏ | ❏ | ❏ | ❏ | ❏ |

What specific elements of poster presentation did you find useful? What should be improved?

|  |
| --- |

The next few statements refer to the computational aspects of the courses (bioinformatics, databases, literature searches, docking, etc.). Please rate how much you agree/disagree with the following statements:

|  | Strongly Disagree | Disagree | Agree | Strongly Agree | Not Sure |
| --- | --- | --- | --- | --- | --- |
| The computational aspects of the courses helped me become more independent in my research. | ❏ | ❏ | ❏ | ❏ | ❏ |
| The computational aspects of the courses made me more confident in my research. | ❏ | ❏ | ❏ | ❏ | ❏ |
| The computational aspects of the courses provided tools for me to be an active participant in discovery. | ❏ | ❏ | ❏ | ❏ | ❏ |
| The computational aspects of the course made my research more tangible. | ❏ | ❏ | ❏ | ❏ | ❏ |
| Overall, I have a deeper understanding of biochemistry due to the computational aspects of these courses. | ❏ | ❏ | ❏ | ❏ | ❏ |

What specific computational aspects of these courses did you find useful? What could be improved?

|  |
| --- |

The next few statements refer to the course lectures/class-times. Please rate how much you agree/dis-agree with the following statements:

|  | Strongly Disagree | Disagree | Agree | Strongly Agree | Not Sure |
| --- | --- | --- | --- | --- | --- |
| I attended lectures regularly. | ❏ | ❏ | ❏ | ❏ | ❏ |
| The lectures worked well in conjunction with the lab. | ❏ | ❏ | ❏ | ❏ | ❏ |
| The lectures were clear and coherent. | ❏ | ❏ | ❏ | ❏ | ❏ |
| The lectures helped me to start thinking independently. | ❏ | ❏ | ❏ | ❏ | ❏ |
| Overall, I have a deeper understanding of biochemistry due to the course lectures. | ❏ | ❏ | ❏ | ❏ | ❏ |

What specific elements of the lectures in the courses did you find useful? What could be improved?

|  |
| --- |

The next few statements will refer to the course labs. Please rate how much you agree/disagree with the following statements:

|  | Strongly Disagree | Disagree | Agree | Strongly Agree | Not Sure |
| --- | --- | --- | --- | --- | --- |
| The labs increased my factual knowledge. | ❏ | ❏ | ❏ | ❏ | ❏ |
| The labs increased my critical thinking. | ❏ | ❏ | ❏ | ❏ | ❏ |
| I have retained skills in experimental design because of the labs. | ❏ | ❏ | ❏ | ❏ | ❏ |
| These labs have given me confidence in my research. | ❏ | ❏ | ❏ | ❏ | ❏ |
| I have retained skills in data analysis because of the labs. | ❏ | ❏ | ❏ | ❏ | ❏ |
| I have retained skills in group work because of the lab. | ❏ | ❏ | ❏ | ❏ | ❏ |
| Overall, I have a deeper understanding of biochemistry due to the course labs. | ❏ | ❏ | ❏ | ❏ | ❏ |

What specific elements of the course laboratories did you find useful? What could be improved?

|  |
| --- |

The next few statements refer to manuscript writing. Please rate how much you agree/disagree with the following statements:

|  | Strongly Disagree | Disagree | Agree | Strongly Agree | Not Sure |
| --- | --- | --- | --- | --- | --- |
| The courses improved my scientific writing skills. | ❏ | ❏ | ❏ | ❏ | ❏ |
| I learned how to organize my research in a scientific manuscript. | ❏ | ❏ | ❏ | ❏ | ❏ |
| Writing the manuscript gave me more confidence in my research. | ❏ | ❏ | ❏ | ❏ | ❏ |
| Overall, I have a deeper understanding of biochemistry due to the introduction of manuscript writing to these courses. | ❏ | ❏ | ❏ | ❏ | ❏ |

What specific elements of the manuscript writing in the courses did you find useful? What could be improved?

|  |
| --- |

The next few statements refer to group meetings. Please rate how much you agree/disagree with the following statements:

|  | Strongly Disagree | Disagree | Agree | Strongly Agree | Not Applicable |
| --- | --- | --- | --- | --- | --- |
| I believe there were a sufficient amount of group meetings. | ❏ | ❏ | ❏ | ❏ | ❏ |
| The group meetings gave me constructive feedback to improve my research. | ❏ | ❏ | ❏ | ❏ | ❏ |
| The group meetings gave me constructive feedback for the final projects of the course (poster and manuscript). | ❏ | ❏ | ❏ | ❏ | ❏ |
| Overall, I have a deeper understanding of biochemistry due to my participation in group meetings. | ❏ | ❏ | ❏ | ❏ | ❏ |

What specific elements of the group meetings in the courses did you find useful? What could be improved?

|  |
| --- |

Please rate how much you agree/disagree with the following statements:

*“Compared to other undergraduate laboratory classes I have taken, this class......*

|  | Strongly Disagree | Disagree | Agree | Strongly Agree | Not Sure |
| --- | --- | --- | --- | --- | --- |
| …encourages more independent thinking. | ❏ | ❏ | ❏ | ❏ | ❏ |
| …teaches more skills in time management. | ❏ | ❏ | ❏ | ❏ | ❏ |
| …teaches more skills in scientific communication. | ❏ | ❏ | ❏ | ❏ | ❏ |
| …better prepares students to present scientific information. | ❏ | ❏ | ❏ | ❏ | ❏ |
| …encourages greater confidence in a student’s scientific knowledge. | ❏ | ❏ | ❏ | ❏ | ❏ |

Please rate how much you agree or disagree with these statements:

|  | Strongly Disagree | Disagree | Agree | Strongly Agree | Not Sure |
| --- | --- | --- | --- | --- | --- |
| I learned to communicate well with my group. | ❏ | ❏ | ❏ | ❏ | ❏ |
| I learned to work professionally with my group. | ❏ | ❏ | ❏ | ❏ | ❏ |
| My group was able to delegate tasks well. | ❏ | ❏ | ❏ | ❏ | ❏ |
| My group met a sufficient amount of time outside of class. | ❏ | ❏ | ❏ | ❏ | ❏ |
| I have a deeper understanding of biochemistry due to working in groups. | ❏ | ❏ | ❏ | ❏ | ❏ |

For the purposes of these courses, I believe the ideal group size would be ______ students.

1. two
2. three
3. four
4. five
5. six
6. seven, or more

Please rank the following items in relation to each other from 1 to 9, with a ‘1’ indicating the greatest contribution to your understanding of biochemistry and a ‘9’ indicating the least contribution.

| Instructor | ❏ |
| --- | --- |
| TA | ❏ |
| Group members | ❏ |
| Assigned readings | ❏ |
| Course Lecture | ❏ |
| Course Lab | ❏ |
| Group meetings | ❏ |
| Poster presentation | ❏ |
| Writing a manuscript | ❏ |

Please briefly elaborate on what you ranked in the above list as having the greatest contribution (‘1’) and what factor you ranked as having the least contribution (‘9’) to your understanding of biochemistry.

|  |
| --- |

Do you feel that these Chem4411/21 courses adequately prepared you to work more independently in a laboratory setting? If so, how? If not, what would have helped you feel more prepared?

|  |
| --- |

Do you feel that these courses helped you think like a scientist rather than a student? If so, how? If not, how can these courses be improved in that respect?

|  |
| --- |

What are the most positive and most negative differences that you saw in these labs, compared to previous lab courses that you have taken? Please elaborate.

|  |
| --- |

These next few statements will refer to your TA in the Chem4421 course (second semester). Please rate how much you agree/disagree with the following statements:

|  | Strongly Disagree | Disagree | Agree | Strongly Disagree | Not Sure |
| --- | --- | --- | --- | --- | --- |
| My TA was able to answer my questions in lab. | ❏ | ❏ | ❏ | ❏ | ❏ |
| My TA was available for questions during their specified office hours. | ❏ | ❏ | ❏ | ❏ | ❏ |
| My TA gave constructive feedback. | ❏ | ❏ | ❏ | ❏ | ❏ |
| My TA was approachable. | ❏ | ❏ | ❏ | ❏ | ❏ |
| Overall, I have a deeper understanding of biochemistry due to the contributions of my TA. | ❏ | ❏ | ❏ | ❏ | ❏ |

What do you think your TA did well? What could your TA improve upon?

|  |
| --- |

Do you have any other comments about these courses?

|  |
| --- |
