# Supplementary material for: Known structure, unknown function: An inquiry‐based undergraduate biochemistry laboratory course
Source: Biochem Mol Biol Educ. 2015 Jul 6;43(4):245–62. doi: 10.1002/bmb.20873 (PMC4758391; doi:10.1002/bmb.20873)
Supplement: Supplementary file 8 — Supporting Information [file BMB-43-245-s008.docx]

Known Structure, Unknown Function:

An Inquiry-based Undergraduate Biochemistry Lab Course

Cynthia Gray, Carol W. Price, Christopher T. Lee, Alison H. Dewald, Matthew A. Cline,

Charles E. McAnany, Linda Columbus, Cameron Mura

Supplementary Information: Overview of Contents

| Supp Info 1: | Precise learning gains, organized by course modules (pp 1–5) |
| --- | --- |
| Supp Info 2: | A sample PyMOL-based in-class activity – Molecular visualization & structural analysis of serine proteases (pp 1–4) |
| Supp Info 3: | Molecular docking tutorial for the Biochemistry Lab (Chem4411/21; pp 1–12) |
| Supp Info 4: | Sample effort report (p 1) |
| Supp Info 5: | Sample grading rubric from the first term (pp 1–3) |
| Supp Info 6: | Sample student assessment of their learning gains (SALG) survey questions (pp 1–4) |
| Supp Info 7: | Sample post–course survey questions (pp 1–8) |
